# Functional Monomers Equipped Microgel System for Managing Parkinson's Disease by Intervening Chemokine Axis‐mediated Nerve Cell Communications

**DOI:** 10.1002/advs.202410070

**Published:** 2024-12-25

**Authors:** Lin Jiang, Xu Zhang, Shun Wang, Jiangkuan Zhang, Junyang Chen, Jiachuan Lu, Liting Yao, Weiwei Jin, Nan Li, Qing Li

**Affiliations:** ^1^ College of Life Sciences China Jiliang University Hangzhou 310018 China; ^2^ Department of Neurology The Second Affiliated Hospital of Zhengzhou University Zhengzhou University Zhengzhou 450052 China; ^3^ School of Life Sciences Zhengzhou University Zhengzhou 450001 China

**Keywords:** chemokine axis, functional monomers, microgel systems, neuroinflammations, Parkinson's diseases

## Abstract

The complex pathology of Parkinson's disease (PD) requires comprehensive understanding and multi‐pronged interventions for communication between nerve cells. Despite new developments in nanotechnology in the treatment of PD, in‐depth exploration of their biological effects, in particular, the specific mechanisms of inflammation inhibition are lacking. Herein, using the stable cascade catalysis channel formed by polydopamine (PDA), imidazole groups, and Cu ions, a microgel system comprising functional monomers [superoxide dismutase (SOD) with double bonds, PDA, 2‐methacryloyloxy ethyl phosphorylcholine (MPC), and Cu ions] is proposed for managing PD. The microgel can be efficiently delivered to the brain aided by MPC, after which a multi‐level regulatory strategy targeting neurons and microglia can be initiated. The catalytic activity cascade elicited by SOD and Cu ions can regulate the anti‐inflammatory phenotypic transformation of microglia by relieving oxidative stress. Meanwhile, the dopamine (DA) released from PDA can facilitate DA storage and neurogenesis, inhibiting CX3CL1 release and the CX3CR1 receptor on microglia and further regulating the CX3CL1/CX3CR1‐NF‐κB‐NLRP3 signaling pathway in microglia to inhibit neuroinflammation. Therefore, the proposed microgel delivery system with functional monomers represents a promising therapeutic strategy for managing neuroinflammation and promoting neurogenesis in PD by intervening chemokine axis‐mediated communication between neurons and microglia.

## Graphical abstract

A novel microgel delivery system with functional monomers for managing Parkinson's disease is designed. The proposed microgel system represents a promising therapeutic strategy for managing neuroinflammation and promoting neurogenesis in PD by intervening chemokine axis‐mediated communications between neurons and microglia.

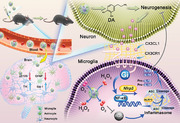

## 1 Introduction

Parkinson's disease (PD) is a gradually worsening neurodegenerative disorder affecting >1% of the population ≥65 years of age, with a prevalence expected to double by 2030.^[^ ^1^ ^]^ The consequences of PD extend beyond individual suffering, leading to considerable economic and societal costs.^[^ ^2^ ^]^ PD is characterized by the degradation of dopaminergic neurons in the substantia nigra pars compacta (SNpc), which is associated with the generation and deposition of α‐synuclein (α‐syn) and neuroinflammation.^[^ ^3^, ^4^ ^]^ Clinically, PD treatment strategies mainly include deep brain stimulation and chemical drugs. Currently, technical barriers limit the application of deep brain stimulation. Therefore, drug therapy is still the primary choice for PD. PD drugs, including levodopa (a dopamine [DA] precursor drug), DA receptor excitation agents, and monoamine oxidase B inhibitors, mainly target DA.^[^ ^5^ ^]^ Nevertheless, during the late treatment stages, the above drugs elicit dyskinesia and psychiatric symptoms because of the loss of dopaminergic neurons.^[^ ^6^, ^7^ ^]^ Therefore, limited drug dosage forms and single treatment strategies are major obstacles limiting the treatment of PD. Thus, it is necessary to develop advanced noninvasive treatments for patients with PD.

To develop efficient therapeutic interventions, understanding the underlying pathogenesis of PD is crucial. The brains of patients with PD are characterized by microglial dysfunction. Microglia are responsible for brain homeostasis by surveying the brain microenvironment to eliminate pathological proteins and other harmful substances; however, overactive microglia exacerbate neuroinflammation caused by reactive oxygen species (ROS).^[^ ^8^, ^9^, ^10^ ^]^ Communications between neurons and microglia play a critical role in the pathology of PD, such as transmission of and interaction between ROS, inflammatory factors, and α‐syn.^[^ ^11^ ^]^ Chemokines are protein ligands that are crucial in the recruitment and activation of immune cells by binding to their cognate chemokine receptors.^[^ ^12^ ^]^ As the only member of the CX3CR subfamily, CX3CR1 mediates the communication between neurons and microglia by recognizing its sole ligand, CX3CL1.^[^ ^13^, ^14^, ^15^ ^]^ In addition, the CX3CL1/CX3CR1 axis participates in numerous human disorders, including neurodegenerative diseases.^[^ ^16^, ^17^ ^]^

Advances in nanomedicine, particularly in the field of drug delivery, immune regulation, and oxidative stress control, have shed light on novel and effective treatment methods for PD.^[^ ^18^, ^19^, ^20^ ^]^ However, despite the development of nanomaterials targeting PD pathogenesis, aiming to protect dopaminergic neurons and regulate the anti‐inflammatory phenotypic transformation of microglia,^[^ ^3^, ^21^ ^]^ nanomaterials that can achieve multifaceted symptom relief of PD by regulating communication between different nerve cells are lacking. In addition, limited blood–brain barrier (BBB) permeation and the potential to cause cell damage because of excessive rigidity restrict the clinical applications of most therapeutic nanomaterials in PD.^[^ ^22^, ^23^, ^24^ ^]^ Hydrogels are polymer nanomaterials that are flexible and modifiable. The advantages of hydrogels in PD treatment are reflected in the following aspects: 1) hydrogels can form 3D scaffolds for the survival and growth of nerve cells.^[^ ^25^ ^]^ 2) hydrogels could encapsulate anti‐PD drugs with various functions, including neuroprotection, inflammation suppression, and nerve growth promotion.^[^ ^26^ ^]^ 3) Functional cells such as dopaminergic cells can be delivered by hydrogels to improve the efficiency of tissue regeneration.^[^ ^27^ ^]^ Further, Microgel systems with satisfactory flexibility and high BBB permeability have been developed.^[^ ^28^ ^]^ For instance, Lu et al. designed several microgels loaded with protein drugs for PD treatment.^[^ ^29^ ^]^ Nevertheless, PD antioxidative treatments rely on ideal charge transfer effects. However, microgels often comprise non‐conductive long carbon chain polymers, which are not conducive to scavenging free radicals through cascade catalysis.^[^ ^30^ ^]^ As a conductive polymer, polydopamine (PDA) is compatible with polymer microgels and can be effectively intercalated into gel to improve the catalytic effects of microgels, especially when coordinated with metal ions.

**Scheme 1:**
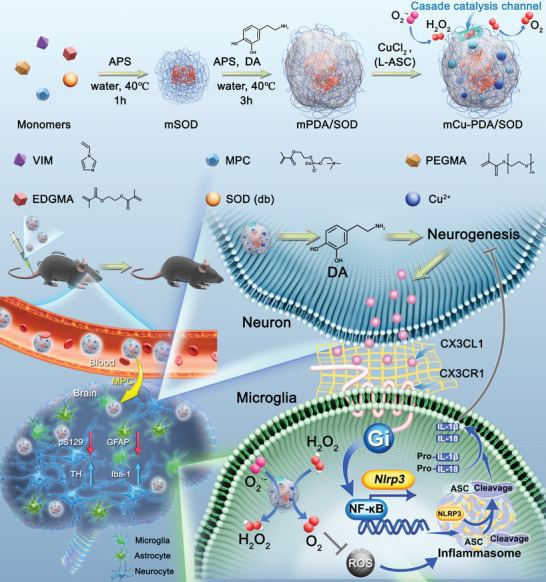
Schematic illustrating the preparation process of mCu‐PDA/SOD microgel system and its therapeutic mechanisms on PD based on promoting neurogenesis and regulating chemokine axis (CX3CL1/CX3CR1)‐mediated communication between neurons and microglia. VIM: vinyl imidazole, APS: ammonium persulphate, DA: dopamine, MPC: 2‐methacryloyloxy ethyl phosphorylcholine, PEGMA: poly(ethylene glycol) methyl ether methacrylate, EDGMA: Ethylene dimethacrylate, SOD (db): Superoxide dismutase with double bonds, pS129: serine 129 phosphorylated α‐synuclein, TH: tyrosine hydroxylase, GFAP: glial fibrillary acidic protein, Iba‐1: ionized calcium‐binding adapter molecule‐1, ROS: reactive oxygen species.

Here, we designed a superoxide dismutase (SOD) and Cu ions‐based microgel system based on multiple functional monomers, which exhibits SOD and catalase (CAT)‐like catalytic activity while promoting neurogenesis and regulating neuroinflammation‐related signaling in PD models. Acetylcholine is widely used for brain targeting and drug delivery due to the presence of acetylcholine receptors in cerebral endothelial vessels. Benefiting from the presence of 2‐methacryloyloxy ethyl phosphorylcholine (MPC) and polyethylene glycol (PEG) groups, the microgel system has an ideal circulation time in vivo, excellent brain targeting, and satisfactory brain enrichment performance due to the acetylcholine group in the MPC. Moreover, PDA and imidazole groups can coordinate with Cu ions, forming a fast and stable cascade catalysis channel for the degradation of H_2_O_2_ produced by SOD catalysis. Notably, an ester bond was used for crosslinking, allowing microgel system degradation for DA release, and further promoting DA replenishment and neurogenesis. Neuronal repair in combination with antioxidant therapy of the microgel system can effectively inhibit the release of chemokines, such as CX3CL1, further regulating the CX3CL1/CX3CR1 axis and the downstream NF‐κB‐NLRP3 pathway, alleviating neuroinflammation in PD models by inhibiting the assembly and formation of the inflammasome (**Scheme** **1**). Therefore, this study aimed to develop a neuron repair and anti‐inflammatory strategy for the treatment of PD, providing new ideas for a multifunctional microgel system regulating nerve cell communication via the CX3CL1/CX3CR1‐NF‐κB‐NLRP3 pathway with potential clinical applications.

## 2 Results and Discussion

### 2.1 Synthesis and Characterization of Microgel Systems

**Figure 1:**
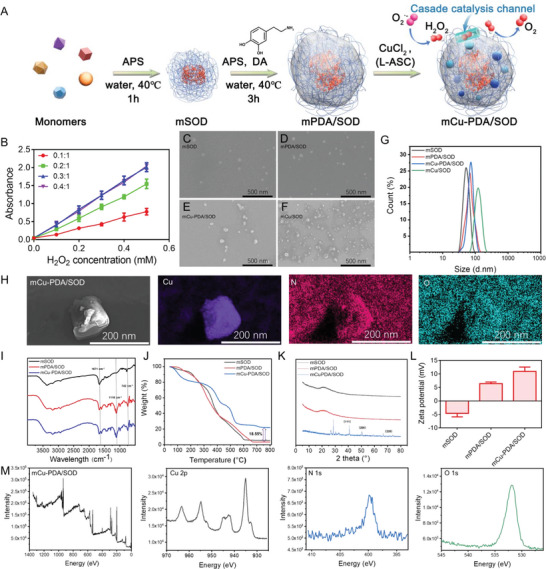
Characterization of microgel systems for successful synthesis. A) Schematic illustrating the preparation process of mCu‐PDA/SOD. B) Catalase (CAT)‐like activity with different Cu ions content (mass ratio: 0.1:1, 0.2:1, 0.3:1, and 0.4:1) of microgels detected by ABTS. *n =* 3 independent experiments. Data represent the mean ± standard deviation (SD); C–F) Scanning electron microscope (SEM) image of mSOD, mPDA/SOD, mCu‐PDA/SOD, and mCu/SOD; G) Particle size of mSOD, mPDA/SOD, mCu‐PDA/SOD, and mCu/SOD in deionized water; H) Mapping images of mCu‐PDA/SOD; I) Fourier transform infrared (FT‐IR) spectra, J) Thermogravimetric analysis (TGA) spectra, K) powder X‐ray diffraction (PXRD) spectra, and L) zeta potential of mSOD, mPDA/SOD, and mCu‐PDA/SOD. *n =* 3 independent experiments. Data represent mean ± SD; M) XPS full and element spectra of mCu‐PDA/SOD.

Microgels were synthesized through radical polymerization (**Figure** **1**A). Double bonds were added on the surface of SOD (SODdb) before adding several monomers to form a SOD‐based microgel (mSOD). During the reaction, DA was added and transformed to PDA in mSOD (mPDA/SOD). Cu^2+^ was coordinated on the mPDA/SOD with the PDA and imidazole groups (mCu‐PDA/SOD). First, the catalase‐like activity with microgels with different Cu^2+^ content (mass ratio: 0.1:1, 0.2:1, 0.3:1 and 0.4:1) were detected by 2, 2′‐azino‐bis(3‐ethylbenzothiazoline‐6‐sulfonic acid, ABTS). As shown in Figure 1B, the absorbance increased with increasing H_2_O_2_ concentration in each group. Microgels displayed the most satisfactory catalase‐like activity when the mass ratio of Cu^2+^:microgel is 0.3:1 or 0.4:1, as the 0.3:1 mass ratio of Cu^2+^:microgel provides a saturated Cu^2+^ ion for coordination. Thus, a 0.3:1 mass ratio of Cu^2+^:microgel was used for the following experiments. Subsequently, the morphologies of microgel were characterized using a scanning electron microscope (SEM), in which mSOD appeared as a ball of nearly 50 nm in diameter (Figure 1C) and mPDA/SOD showed a similar structure to mSOD (Figure 1D). In contrast, Cu ions coordinated with the microgel forming a crystal structure on the microgel surface, and the size of mCu‐PDA/SOD was 60 nm (Figure 1E). Additionally, a microgel without PDA (mCu/SOD) was synthesized, and its size was irregular (Figure 1F). Due to the limited coordination sites, some Cu ions will coordinate with multiple gels, resulting in uneven particle size in the mCu/SOD group. By contrast, DA can provide more coordination sites for Cu ions, and Cu ions are dispersed in a single gel after complete dispersion, so the particle size is controllable and uniform. Moreover, the clearer microgel transmission electron microscopy (TEM) images are shown in Figure S1 (Supporting Information). The particle size in deionized water (DI‐water) detected by dynamic light scattering confirmed these results (Figure 1G). The larger particle size was inappropriate for PD delivery and treatment. Thus, adding PDA to the microgel system is necessary for controlling particle size. In addition, a certain aggregation between microgels could be observed, which is the result of interactions of the hydrogen bonds such as polyethylene glycol and DA. However, the microgel aggregation is weak, and the particle sizes after aggregation are within the acceptable range of biotherapy. Moreover, such aggregation will be weakened due to the decrease in concentration in blood. Therefore, the effect of microgel aggregation in biotherapy is negligible. For Cu ions distribution, the mapping results showed that Cu ions co‐localized with the nitrogen and oxygen in the microgel, confirming that Cu ions were coordinated with the microgel (Figure 1H).

Further characterization by Fourier transform infrared spectroscopy showed the PEG peak (representing the ethylene glycol dimethylacrylate (EDGMA) monomer) at 3100–3500 cm^−1^, confirming successful mSOD synthesis (Figure 1I). The peak at 1671 cm^−1^ was because of the aromatic ring resonance of PDA, and that at 1116 cm^−1^ was formed by the bending vibration of C─H and tensile vibration of the N‐H of the aromatic ring. Meanwhile, the bending vibration of C─H and tensile vibration of C─C appeared at 742 cm^−1^. These three peaks confirmed the successful synthesis of PDA on the microgel, which shifted after adding Cu^2+^, indicating that Cu ions coordinate with PDA on the microgel. Consistently, mCu/SOD did not show the PDA characteristic peaks (Figure S2, Supporting Information). Thermogravimetric analysis was applied to examine the Cu ions content by heating in the air atmosphere. As shown in Figure 1J, there was little difference in weight loss between mSOD and mPDA/SOD. However, at 675 °C, the weight loss stopped, and the mCu‐PDA/SOD showed 18.55% lower weight loss than mPDA/SOD because of the Cu ions content in the microgel. Meanwhile, mCu/SOD showed a 9% Cu content according to the weight loss of mCu/SOD and mSOD, indicating that the coordination and binding of Cu will decrease significantly without PDA (Figure S3, Supporting Information). In addition, PDA is not stable under acidic conditions and can be converted to DA. Due to the protonation of hydrogen ions, acidic conditions are not conducive to Cu ions coordination.

As for the dispersion of Cu ions in microgel, the mCu‐PDA/SOD was incubated in PBS solutions of pH = 5 or pH = 7, and then detected by inductively coupled plasma‐mass spectrometry (ICP‐MS). The acid environment was able to disaggregate the Cu ions at the surface. As shown in Figure S4 (Supporting Information), the Cu ion was ≈18.4% content in the mCu‐PDA/SOD and ≈4.9% Cu of mass ratio was remaining inside of mCu‐PDA/SOD. From the calculation, the Cu ion on the surface accounts for 73.3% of the total Cu ion in mCu‐PDA/SOD. The crystal structure of mCu‐PDA/SOD obtained using powder X‐ray diffraction is shown in Figure 1K. mSOD and mPDA/SOD displayed a sharpness peak with no crystal structure. In contrast, mCu‐PDA/SOD showed several sharp peaks including Cu‐PDA coordination peaks ([111], [200], and [220]) and other peaks similar to mCu/SOD (Figure S5, Supporting Information). Furthermore, mSOD displayed a negative zeta potential. However, after PDA and Cu ions decoration, the potential turned positive, which helps permeate the BBB (Figure 1L). Subsequently, X‐ray photoelectron spectroscopy (XPS) was used to examine the coordination between Cu ions and the microgel (Figure 1M; Figure S6, Supporting Information). Cu ions peaks appeared in the full spectrum of mCu‐PDA/SOD, with Cu^2+^displaying bivalent and monovalent states in the microgel in the XPS spectrogram because of the PDA, imidazole, and oxygen coordinating with Cu ions.

### 2.2 Enzyme‐like Activity of Microgel Systems

**Figure 2:**
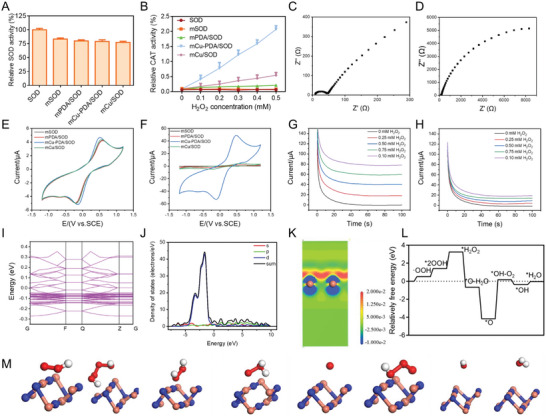
Catalytic activity and mechanism of mCu‐PDA/SOD. Relative A) superoxide dismutase (SOD)‐like activity and B) CAT‐like activity of free SOD, mSOD, mPDA/SOD, mCu‐PDA/SOD, and mCu/SOD. *n =* 3 independent experiments. Data represent mean ± SD; Electrical impedance scanning (EIS) trace of C) mCu‐PDA/SOD and D) mCu/SOD; E) cyclic voltammetry (CV) curves of mSOD, mPDA/SOD, mCu‐PDA/SOD, and mCu/SOD in 0.1 m PBS solution; F) CV curves of mSOD, mPDA/SOD, mCu‐PDA/SOD, and mCu/SOD in 0.1 m PBS with 0.05 m H_2_O_2_ solution; *I*–*t* curves of G) mCu‐PDA/SOD and H) mCu/SOD at different concentrations of H_2_O_2_; I) Binding structure, J) density of states, and K) differential charge density analyses of mCu‐PDA/SOD; L) binding energy and M) binding structure of mCu‐PDA/SOD with reaction intermediate.

UV–vis spectroscopy was used for further detection of SOD and CAT activity. The assembly of the enzyme is bound to lead to a decline in its activity due to the interference of substrate and enzyme contact.^[^ ^31^, ^32^ ^]^ As shown in **Figure** **2**A, mSOD displayed ≈80% relative SOD activity compared with that of free SOD, whereas other microgel systems showed similar SOD‐like activity to mSOD, indicating that the effect of microgel assembly on SOD activity was in an acceptable range and did not significantly change the enzyme activity. Conversely, mCu‐PDA/SOD displayed the best CAT‐like activity at different H_2_O_2_ concentrations due to the coordination effect of Cu ions with PDA, that is, PDA endows Cu with more coordination sites and more coordination forms. mPDA/SOD had a weaker CAT‐like activity than that of mSOD and free SOD (Figure 2B). Moreover, mCu/SOD also showed CAT‐like activity but lower than mCu‐PDA/SOD. This result implied that the presence of PDA on the microgel could provide better CAT‐like activity after Cu ions coordination.

Furthermore, we used electronic detection to examine electronic conduction for catalysis. As shown in Figure 2C,D, electrical impedance scanning detected the resistance of mCu/SOD and mCu‐PDA/SOD. mCu‐PDA/SOD displayed lower resistance (25 Ω) than mCu/SOD. The conducting polymer PDA offered a better electronic transmission to the microgel. Similarly, for cyclic voltammetry in phosphate‐buffered saline (PBS) (Figure 2E), mCu‐PDA/SOD showed narrower spacing between oxidation and reduction peaks than other groups. This implies that mCu‐PDA/SOD possesses a satisfactory oxidation–reduction reaction ability. Furthermore, after adding H_2_O_2_ to PBS, mCu‐PDA/SOD displayed a higher electrochemical response, which confirmed that mCu‐PDA/SOD possesses a superior CAT‐like activity (Figure 2F). Different H_2_O_2_ concentrations were added to investigate the catalytic efficiency by *i*–*t* curves. As shown in Figure 2G,H, both mCu/SOD and mCu‐PDA/SOD had a higher current with increasing H_2_O_2_; however, mCu‐PDA/SOD showed a better response. Moreover, as for the immobilization efficiency, the SOD was totally immobilized in the mCu‐PDA/SOD. Nanodrop was used to detect the SOD content in mCu‐PDA/SOD and the reaction solution. As shown in Figure S7 (Supporting Information), the SOD mass ratio in mCu‐PDA/SOD was calculated ≈18.6%. The reaction solution was extracted, and hardly any SOD was detected.

The catalytic mechanism of mCu‐PDA/SOD was detected by density functional theory. Based on the band structure of mCu‐PDA/SOD in Figure 2I, an indirect band gap of 0.089 eV appeared and the Fermi level was closer to the valence band maximum, indicating that the holes which readily accepted electrons were mostly responsible for the biocatalytic activity of mCu‐PDA/SOD. For the energy level principle, mCu‐PDA/SOD's partial and total density of states were calculated, which revealed that valence and conduction bands mainly comprised Cu^2+^ d orbitals (Figure 2J). Thus, the electron structure of mCu‐PDA/SOD was mostly determined by the d‐band center. Differential charge density analyses were conducted (Figure 2K), with red and blue colors indicating electron accumulation and depletion, respectively. There was obvious electron aggregation around mCu‐PDA/SOD. Furthermore, the binding energy of the catalysis procedure is shown in Figure 2L,M, which confirms that H_2_O_2_ and the reaction intermediate tend to combine with mCu‐PDA/SOD.

### 2.3 Protection of Microgel Systems Against Neuroinflammation and Apoptosis In Vitro

**Figure 3:**
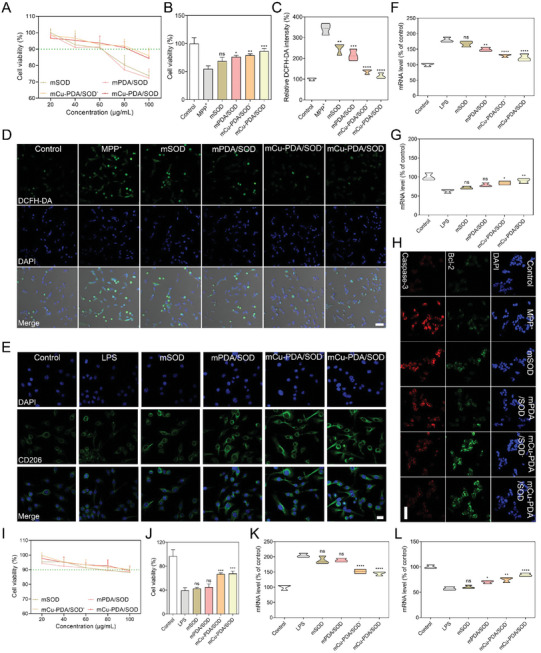
Neurocellular protection of microgel systems in vitro. A) Cell viability of SH‐SY5Y cells exposed to microgel systems with various concentrations, *n =* 3 independent experiments. Data represent the mean ± SD. B) Cell viability of SH‐SY5Y cells exposed to MPP^+^ following treatment with microgel systems, *n =* 3 independent experiments. Data represent the mean ± SD. Intracellular ROS detection using DCFH‐DA as a probe after treatment with microgel systems under the stimulation of MPP^+^. C) Quantitative results of probe fluorescence intensity and D) the corresponding fluorescence detection images, *n =* 3 independent experiments. Data represent the mean ± SD. The scale bar is 50 µm. E) Immunofluorescence staining of CD206 in BV2 cells. The scale bar is 20 µm. The expression levels of F) typical proinflammatory cytokines (IL‐6) and G) anti‐inflammatory factors (IL‐10) in BV2 cells. *n =* 3 independent experiments. Data represent the mean ± SD. H) Cell apoptosis detection via immunofluorescence staining of bcl‐2 and caspase‐3. The scale bar is 50 µm. I) Cell viability of microglia isolated from mice and exposed to microgel systems with various concentrations, *n =* 3 independent experiments. Data represent the mean ± SD. J) Cell viability of isolated microglia exposed to LPS following treatment with microgel systems, *n =* 3 independent experiments. Data represent the mean ± SD. The expression levels of K) typical proinflammatory cytokines (IL‐6) and L) anti‐inflammatory factors (IL‐10) in isolated microglia. *n =* 3 independent experiments. Data represent the mean ± SD. The statistical analyses were conducted using GraphPad Prism 8.0.2. The outcomes were compared via one‐way ANOVA (with Tukey's post hoc correction for multiple comparisons). *p < 0.01, **p < 0.005, ***p < 0.001, ****p < 0.0001, ns, not significant.

Human neuroblastoma cells (SH‐SY5Y) and mouse microglia (BV2) were selected as model cells for toxicity analysis. As shown in **Figures** **3**A, and S8 (Supporting Information), these microgel systems exhibited negligible toxicity in the above two cell lines for doses <60 µg mL^−1^; thus, this dose was selected for subsequent experiments. Subsequently, a PD‐like phenotype was induced in SH‐SY5Y cells under 1‐methyl‐4‐phenylpyridinium (MPP^+^) stimulation and the effects of various preparations on the PD‐like phenotype were determined by an MTT assay. As shown in Figure 3B, microgel systems dramatically increased the cell viability suppressed by MPP^+^ stimulation, where mCu‐PDA/SOD exhibited the most significant effect. Next, we investigated the relationship between the effect of microgels on cell viability and their antioxidant activity, showing that mCu‐PDA/SOD^−^ (mCu‐PDA/SOD without acetylcholine) and mCu‐PDA/SOD significantly decreased DCFH‐DA fluorescence in SH‐SY5Y cells, indicating the removal of redundant ROS (Figure 3C,D). M2‐like phenotype microglia exert anti‐inflammatory effects by releasing cytokines in response to ROS overproduction.^[^ ^4^ ^]^ Immunofluorescence images and the corresponding quantitative analysis of CD206, a biomarker of M2‐like phenotype microglia, indicated that mCu‐PDA/SOD treatment significantly increased the expression of CD206 in BV2 cells (Figure 3E; Figure S9, Supporting Information).

In addition, the expression of typical proinflammatory cytokines (IL‐6) and anti‐inflammatory factors (IL‐10) in the conditioned media of BV2 cells was determined. As shown in Figure 3F,G, mCu‐PDA/SOD treatment decreased IL‐6 levels and increased those of IL‐10, having higher therapeutic efficiency than other microgel systems. Furthermore, as an important avenue of cell death induced by ROS, the apoptosis of SH‐SY5Y cells and the inhibitory effect of microgel systems on apoptosis were detected. The results in the immunofluorescence assay demonstrated that treatment with mCu‐PDA/SOD notably decreased the expression of the pro‐apoptotic protein caspase‐3 while increasing that of the anti‐apoptotic protein bcl‐2 (Figure 3H). Further, primary cells partially isolated from mice brains were used to further verify the role of mCu‐PDA/SOD. As shown in Figure 3I, in the concentration range of 20–60 µg mL^−1^, the toxicity of mCu‐PDA/SOD to cells is negligible. Meanwhile, mCu‐PDA/SOD significantly protected primary cells from lipopolysaccharide (LPS) stimulation (Figure 3J), reducing IL‐6 expression (Figure 3K), and promoting IL‐10 expression (Figure 3L), which is consistent with the results in BV2 cells.

### 2.4 PD Lesion‐Specific Targeting of the Microgel Systems and Behavioral Evaluation After Therapy

**Figure 4:**
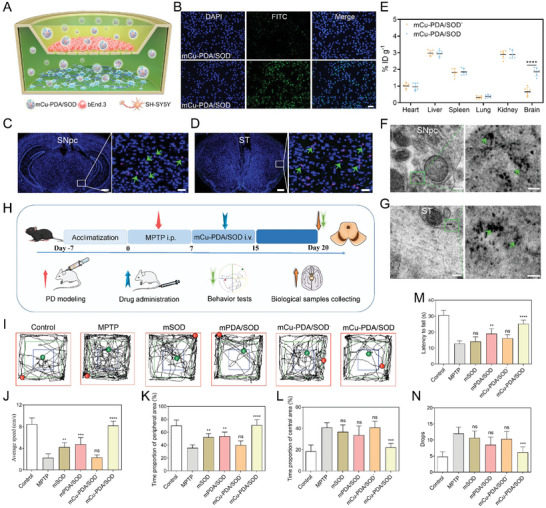
Brain enrichment and behavioral testing in PD mice. A) Schematic illustrating the BBB model in a transwell assay. B) The fluorescence microscope images of FITC‐labeled microgel systems in the SH‐SY5Y cells in a transwell assay. The scale bar is 50 µm. The brain enrichment in the C) SNpc and D) ST regions of fluorescent‐labeled mCu‐PDA/SOD. The scale bars are 500 µm in the original images and 50 µm in the enlarged images. E) Biodistribution of mCu‐PDA/SOD^−^ and mCu‐PDA/SOD in the brain and major organs via ICP‐MS, *n =* 6 independent animals. Data represent the mean ± SD. Bio‐TEM images showing the distribution of mCu‐PDA/SOD in the F) SNpc and G) ST of the mice brain. The scale bars are 200 nm in the original images and 50 nm in the enlarged images. H) Schematic demonstrating the schedules of establishment, microgel systems therapy, and biological sample handling of PD mice. I) The representative path tracing of mice in the open field test, and quantitative analysis of J) average speed, K) time proportion of peripheral area, L) time proportion of the central area of mice. Investigations of the main indicators include M) latency to fall and N) drops in the rotarod test, *n =* 6 independent animals. Data represent the mean ± SD. The statistical analyses were conducted using GraphPad Prism 8.0.2. The outcomes were compared via one‐way ANOVA (with Tukey's post hoc correction for multiple comparisons). **p < 0.005, ***p < 0.001, ****p < 0.0001, ns, not significant.

As a selective penetration barrier between the brain and blood, the BBB is crucial in maintaining the normal cerebral physiological state; it also controls the uptake of most PD therapeutic drugs by the brain.^[^ ^23^ ^]^ To evaluate the specific targeting capability of microgel systems with/without acetylcholine, an in vitro BBB model was constructed, wherein SH‐SY5Y cells and brain endothelial (bEnd.3) cells were co‐cultured in a transwell cella to simulate the BBB.^[^ ^3^, ^33^ ^]^ In addition, fluorescent dye fluorescein isothiocyanate (FITC)‐labeled microgel systems were added into the upper chamber of the transwell cella (**Figure** **4**A). Figure 4B and Figure S10A, (Supporting Information) show that after, incubation for 24 h, FITC‐mCu‐PDA/SOD was transported across the BBB and into the lower chamber and internalized by SH‐SY5Y cells with higher efficiency than FITC‐mCu‐PDA/SOD^−^. Further, ICP‐MS results also indicated that there were more mCu‐PDA/SOD transported across the BBB than mCu‐PDA/SOD^−^ (Figure S11, Supporting Information). Moreover, the trans‐endothelial electrical resistance of the BBB simulated by bEnd.3 did not obviously change during the 24 h of microgel treatment, which proved that the treatment would not destroy the integrity of the BBB (Figure S10B, Supporting Information).

Subsequently, panoramic section scanner imaging was used to observe the distribution of Cy5.5‐labeled mCu‐PDA/SOD in brain tissues. PD mouse models were intravenously injected with Cy5.5‐mCu‐PDA/SOD. After 12 h, mice were sacrificed and their brains were used to prepare paraffin sections and subjected to fluorescence or bio‐TEM imaging. Figure 4C,D shows significant fluorescence in the SNpc and striatum (ST) after treatment. Additionally, bio‐TEM illustrated a clear distribution of mCu‐PDA/SOD in the SNpc (Figure 4F) and ST (Figure 4G), indicating that acetylcholine can significantly improve brain targeting and increase the mCu‐PDA/SOD content in the brain lesion. Furthermore, ICP‐MS was performed to investigate the biodistribution of microgel systems in major organs. Mice were sacrificed and their organs (heart, liver, spleen, lung, kidney, and brain) were collected 24 h after injection of the microgel systems. Furthermore, the tissues were weighed and homogenized to calculate the injected dose per gram of tissue (% ID g^−1^). Figure 4E shows that mCu‐PDA/SOD exhibited satisfactory normalized dosage accumulation with 1.86% ID g^−1^ in the brain, while only 0.66% ID g^−1^ accumulated in the brain after mCu‐PDA/SOD^−^ treatment. Specifically, mCu‐PDA/SOD showed more accumulations in the SNpc and ST (Figure S12, Supporting Information) regions compared with those of mCu‐PDA/SOD^−^, and the content in the SNpc/ST region made up ≈20% of the total mCu‐PDA/SOD in the brain. Combined with the above results in vivo and in vitro, we considered that mCu‐PDA/SOD can permeate the BBB highly efficiently, targeting the brain and producing SNpc/ST accumulation with the assistance of acetylcholine. In addition, in order to provide evidence on how long mCu‐PDA/SOD remain in the brain and explain how they are cleared from the brain and body, we performed ICP‐MS experiments to analyze the concentration of mCu‐PDA/SOD in the brain, liver, and kidney at different time after administration (Figure S13, Supporting Information). The results suggested that mCu‐PDA/SOD accumulated rapidly in the brain and reached a peak at 24 h after administration. At 36 h, the level of mCu‐PDA/SOD decreased to the starting point. By contrast, it maintained at a high level in the liver and kidney from 24 to 36 h after administration, suggesting that mCu‐PDA/SOD was metabolized by the liver and kidneys, just like most other intravenous medications.

As shown in Figure 4H, the motor function and exploration ability in PD mice were evaluated using a series of behavioral tests including the open field, rotarod, and pole‐climbing tests after the administration of microgel systems. mCu‐PDA/SOD‐treated mice traveled faster across the open field than PD mice (Figure 4J). The trajectories of PD mice concentrated in the central area; however, after treatment with mCu‐PDA/SOD, the movement area got relatively focused on the periphery, indicating a strong exploratory ability in the open field test (Figure 4I,K,L). In addition, the rotarod test was used to evaluate balance and coordination with two indicators, total drops and latency to fall. Figure 4M,N shows that, compared with PD mice, the latency to fall significantly increased from 12.8 to 25.2 s while the number of drops decreased from 12.0 to 6.2 after mCu‐PDA/SOD treatment. Similar results were observed in the pole test, which served to assess bradykinesia based on time to turn (T‐turn) and time to reach the bottom (T‐total). As shown in Figure S14A,B (Supporting Information) compared to PD mice, mCu‐PDA/SOD‐treated mice showed lower T‐turn and T‐total, while the administration of other microgel systems did not significantly decrease any of the two indexes.

### 2.5 Pathological Evaluations in PD Mice After Treatment With Microgel Systems

**Figure 5:**
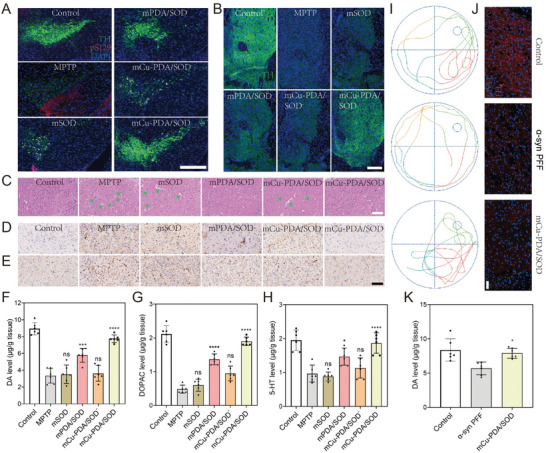
Pathological assessments in PD mice. Co‐immunoreactivity analysis of major pathological factors including pS129 and TH in the A) SNpc and B) ST regions of the PD mice brain. The scale bars are 400 µm in figures A and B. C) Hematoxylin & eosin (H&E) staining of the SNpc tissues in PD mice after microgel systems treatment. The scale bar is 100 µm. D) GFAP and E) Iba‐1 immunohistochemical staining of the SNpc tissues in PD mice. The scale bar is 100 µm. The levels of F) DA, G) DOPAC, and H) 5‐HT in the ST of mice brains, *n =* 6 independent animals. Data represent the mean ± SD. I) The path tracing of mice in the Morris water maze test used to assess the behavior of α‐syn PFF‐induced PD mice after treatment with mCu‐PDA/SOD. J) Immunoreactivity analysis of TH in the ST region of α‐syn PFF‐induced PD mice. The scale bar is 50 µm. K) The levels of DA in the ST region of mice, *n =* 6 independent animals. Data represent the mean ± SD. The statistical analyses were conducted using GraphPad Prism 8.0.2. The outcomes were compared via one‐way ANOVA (with Tukey's post hoc correction for multiple comparisons). *p < 0.01, ***p < 0.001, ****p < 0.0001, ns, not significant.

Loss of TH^+^ neurons in the SNpc and ST is characteristic of PD pathology, as are the decreased levels of DA and its metabolites, including DOPAC and 5‐TH. In addition, α‐syn accumulation has been widely used as a marker to determine PD severity, which can aggravate TH^+^ neuron loss and decrease DA levels.^[^ ^34^ ^]^ Serine 129 phosphorylated α‐syn (pS129) has been used as a biomarker to assess the degree of α‐syn accumulation and pathology. Thus, co‐immunofluorescence for TH and pS129 was performed in the SNpc, which suggested that the pS129 level significantly increased while that of TH decreased in MPTP‐induced PD mice. In contrast, mCu‐PDA/SOD significantly reversed the situation compared to other hydrogel systems (**Figure** **5**A; Figure S15, Supporting Information). Further, we investigated TH changes in the ST of the different experimental groups (Figure 5B); mCu‐PDA/SOD‐treated mice exhibited similar TH levels to those of the control group.

Next, the brain tissues were subjected to cell morphology analysis to determine the degree of damage to neurons. As shown in Figure 5C, many neurons in the brain tissues of PD mice were hypochromic with pyknosis, with an unclear boundary between the cytoplasm and nucleus and an irregular cell shape; these effects were substantially ameliorated after mCu‐PDA/SOD treatment. The excessive number of activated astrocytes and microglia are other pathological features of PD, which can exacerbate neuroinflammation and depletion of dopaminergic neurons.^[^ ^35^ ^]^ The results illustrated that PD mice showed more GFAP (an astrocyte marker; Figure S16A, Supporting Information) and Iba‐1 (a microglial marker; Figure S16B, Supporting Information) immunogenicity in the SNpc, which were significantly reduced after treatment with mCu‐PDA/SOD. Consistently, ELISA assays found significantly higher content of striatal DA (Figure 5F), dihydroxyphenylacetic acid (Figure 5G), and 5‐hydroxytryptamine (Figure 5H) levels in mCu‐PDA/SOD‐treated mice, and comparable to those of control mice. Altogether, these results illustrate that mCu‐PDA/SOD can reduce the content of pathologic protein and the presence of glial cells, while increasing TH expression in the SNpc and ST, thus ameliorating the neurotoxic effect of MPTP and pathological symptoms of PD mice. Since increased neuroinflammation is a comprehensive manifestation of PD symptoms, neuroinflammation is significantly increased in the brain with PD disease.^[^ ^2^, ^3^ ^]^ Therefore, the relief of neuroinflammation contributes to the mitigation of PD symptoms. Meanwhile, mCu‐PDA/SOD has ideal brain targeting performance and can specifically accumulate into the brain region, while producing negligible side effects on other major organs (Figure S17, Supporting Information). Additionally, the contents of immunoglobulin (Ig)G, and IgA, as well as complement (C)3 and C4 did not clearly change at different times after administration (Figure S18, Supporting Information), indicating that mCu‐PDA/SOD will not elicit remarkable immunogenic response in vivo, proving its low biotoxicity and promise in future clinical transformation.

Further, a‐syn preformed fibrils (a‐syn PFF)‐induced PD mice were applied for further verification, which suggested that mCu‐PDA/SOD could improve the behavioral disorders and increase the levels of TH and DA in the ST region of PD mice (Figure 5I–K). However, in MPTP‐induced PD mice, mCu‐PDA/SOD exhibited higher efficiency in alleviating the behavioral and pathological symptoms of PD, so MPTP‐induced PD mice were selected for subsequent studies.

### 2.6 Biological Effects and Therapeutic Mechanisms of mCu‐PDA/SOD

**Figure 6:**
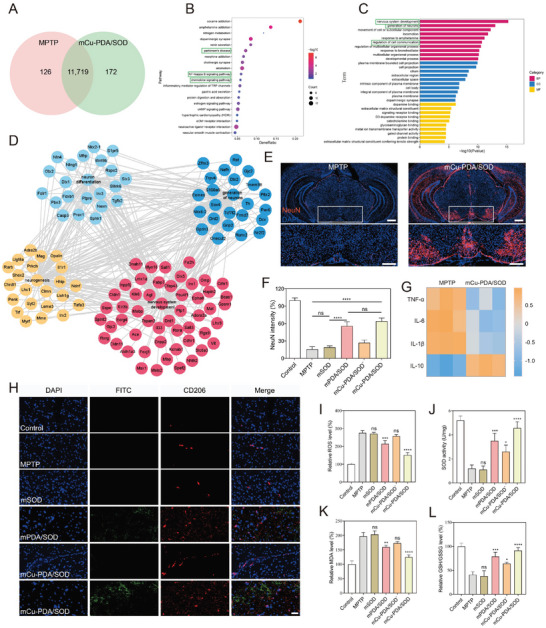
Transcriptomic analysis and verification on the biological effects of microgel systems in PD models. A) Venn diagram of transcriptomic profiles on MPTP‐induced PD mice with or without mCu‐PDA/SOD treatment. B) KEGG pathway enrichment and C) GO enrichment analysis of the identified differentially expressed genes DEGs. The boxes circled the interested signaling pathways. D) Biological process in GO analysis showing the differentially expressed pathways in PD mouse models with or without mCu‐PDA/SOD treatment. E) Immunofluorescence staining of NeuN in the SNpc of PD mice with or without mCu‐PDA/SOD treatment. The scale bars are 500 µm in the original images and 300 µm in the enlarged images. F) Quantitative analysis of NeuN intensity in the SNpc of PD mice, *n =* 6 independent animals. Data represent the mean ± SD. G) Heat map of inflammatory factors expression in the SNpc of PD mice brain with or without mCu‐PDA/SOD treatment. H) Immunofluorescence staining of CD206 in the SNpc of PD mice after treatment with microgel systems. The scale bar is 40 µm. I) ROS, J) SOD activity and K) MDA content, and L) GSH/GSSG in the brains of PD models after treatment with microgel systems, *n =* 3 independent animals. Data represent the mean ± SD. The statistical analyses were conducted using GraphPad Prism 8.0.2. The outcomes were compared via one‐way ANOVA (with Tukey's post hoc correction for multiple comparisons). *p < 0.01, **p < 0.005, ***p < 0.001, ****p < 0.0001, ns, not significant.

Subsequently, transcriptomic analysis was carried out to illustrate the underlying therapeutic mechanisms of mCu‐PDA/SOD at the gene level. Our unguided analysis of differential expression showed that 11 719 genes were co‐expressed in PD mice with or without mCu‐PDA/SOD treatment, compared with 172 genes exclusively expressed in mCu‐PDA/SOD‐treated PD mice (**Figure** **6**A). Kyoto Encyclopedia of Genes and Genomes enrichment analysis showed a remarkable change in gene expression in the PD, with NF‐kappa B (NF‐κB) and chemokine signaling pathways, highly related to the therapeutic mechanisms of mCu‐PDA/SOD (Figure 6B). Further, Gene Ontology (GO) enrichment analysis (Figure 6C,D) showed significant differences in nervous system development, neurogenesis, and neuron differentiation after treatment with mCu‐PDA/SOD. To confirm the specific mechanisms identified via GO enrichment analysis, a NeuN immunofluorescence assay in the SNpc indicated increased NeuN immunogenicity after mCu‐PDA/SOD treatment compared to PD mice (Figure 6E,F), further confirming the effects of mCu‐PDA/SOD in promoting neurogenesis.

ROS activates the NF‐κB signaling pathway, inducing a systemic inflammatory response.^[^ ^36^ ^]^ Thus, we evaluated the efficacy of mCu‐PDA/SOD in controlling the neuroinflammation induced by oxidative damage. Neuroinflammation, the crucial hallmark in the occurrence and development of PD, is characterized by decreased antioxidative capacity, inflammatory cytokine release, and phenotypic changes in microglia. As resident macrophages in the brain, microglia benefit brain homeostasis by removing cell debris and pathogens, as well as providing nutritional factors for the brain. However, overactive microglia can promote ROS production and proinflammatory cytokine release, which can cause or aggravate the pathogenesis of PD.^[^ ^37^ ^]^ As shown in Figure 6G, the levels of proinflammatory cytokines including tumor necrosis factor‐α (TNF‐α), IL‐6, and IL‐1β in PD models were higher than those in the control group; an effect reversed upon mCu‐PDA/SOD treatment. In contrast, the level of anti‐inflammatory cytokines such as IL‐10 was increased after mCu‐PDA/SOD administration, suggesting a mitigating effect on neuroinflammation of mCu‐PDA/SOD. Next, the therapeutic mechanisms of mCu‐PDA/SOD regarding microglia activation and neuroinflammation were evaluated. First, immunofluorescence staining for CD206 and CD86 (a marker of proinflammatory M1 microglia) were performed; the results indicated that M2 microglia gradually increased when microgel systems entered the brain parenchyma (Figure 6H; Figures S19 and S20, Supporting Information). Additionally, ROS and antioxidant capacity in the brain SNpc were also significantly altered after mCu‐PDA/SOD treatment, which can be evidenced by the decreased ROS and malondialdehyde levels, and elevated GSH/GSSG and SOD activity (Figure 6I–L).

### 2.7 Therapeutic Mechanism Related to Nerve Cell Communications

**Figure 7:**
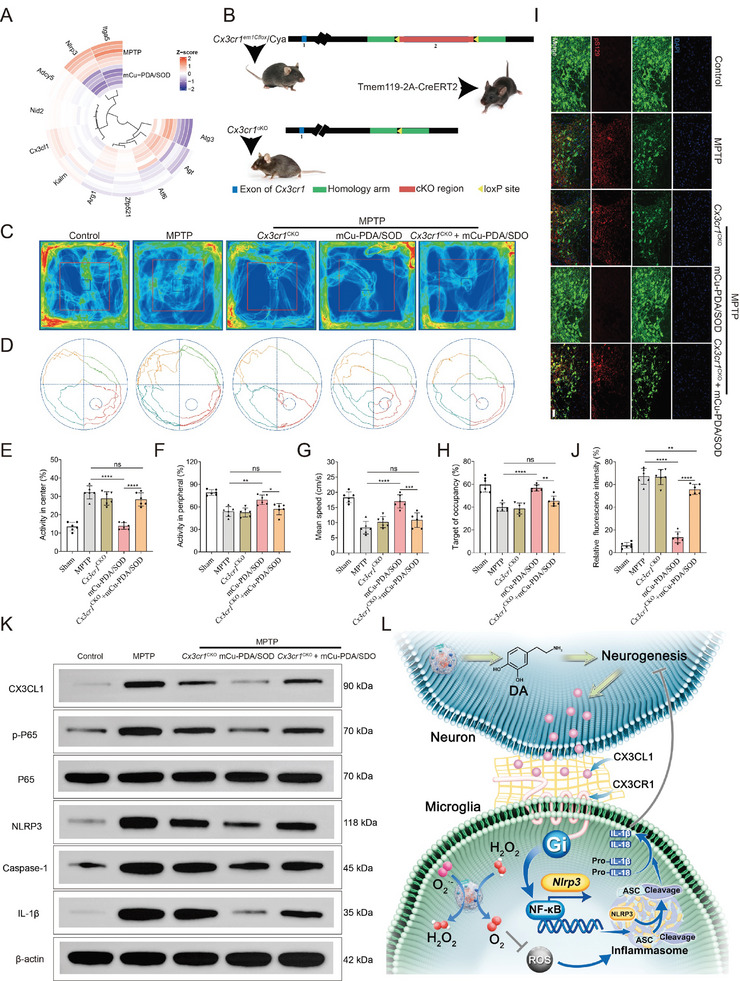
Analyses of the biotic effect mechanisms concerning mCu‐PDA/SOD acting on the chemokine axis‐mediated cellular communication in PD models. A) Heat map of transcriptomics analysis showing the genes that are differentially expressed before and after mCu‐PDA/SOD treatment. B) Schematic diagram illustrating the breeding process of *Cx3cr1* ^CKO^ mice using the *Cre‐Loxp* recombinant enzyme system, in which the C57BL/6‐*Cx3cr1^em1Cflox^*/Cya mice and microglia‐specific Cre mice (Tmem119‐2A‐CreERT2) were bred. The representative moving paths of PD mice and *Cx3cr1* ^CKO^ mice with or without mCu‐PDA/SOD treatment in the C) open‐filed test and the D) Morris water maze test. The main indicators in the open field test include E) activity in the center and F) activity in the periphery, and in the Morris water maze test including G) mean speed and H) target of occupancy, *n =* 6 independent animals. Data represent the mean ± SD. I) Co‐immunoreactivity analysis of the brain sections from PD mice by immunofluorescence, stained with anti‐pS129 antibody (red) and anti‐TH antibody (green). The scale bars are 50 µm. J) The corresponding quantitative results expressed as the relative fluorescence intensity of pS129 and TH, *n =* 6 independent experiments of independent animals. Data represent the mean ± SD. K) Western blotting assay showing the activation of CX3CL1/CX3CR1‐NF‐κb pathway and assembly of NLRP3 inflammasome in the SNpc of PD mice and *Cx3cr1* ^CKO^ mice with or without mCu‐PDA/SOD treatment. L) Schematic illustrating the action mechanism of mCu‐PDA/SOD in regulating CX3CL1/CX3CR1‐mediated NF‐κb signaling pathway and the assembly of NLRP3 inflammasome, thus alleviating neuroinflammation and promoting neurogenesis in PD treatment. The statistical analyses were conducted using GraphPad Prism 8.0.2. The outcomes were compared via one‐way ANOVA (with Tukey's post hoc correction for multiple comparisons). *p < 0.01, **p < 0.005, ***p < 0.001, ****p < 0.0001, ns, not significant.

Neuroinflammation is associated with cytokine and chemokine release, in which cell communication, especially the crosstalk between neurons and microglia, plays an irreplaceable role.^[^ ^38^ ^]^ The franctalkine (CX3CL1)/CX3CR1 axis is the most relevant involving neurons and microglia. The ligand CX3CL1 is expressed and secreted by neurons while its receptor CX3CR1 is in microglia.^[^ ^39^ ^]^ Considering the RNAseq results, *Cx3cl1*, *Nlrp3* (**Figure** **7**A), and the NF‐κB pathway (Figure 6B) are key components explaining the therapeutic effects of mCu‐PDA/SOD, we continued to explore the role of the inflammatory pathways mediated by the chemokine axis (CX3CL1/CX3CR1) in the treatment of PD.

First, a conditional knockout of *Cx3cr1* was established in the brain of C57BL/6 mice (*Cx3cr1* ^CKO^) by *Cre‐Loxp* recombination (Figure 7B), to then evaluate the effects of knocking out *Cx3cr1* on those of mCu‐PDA/SOD. Initially, the behavioral ability of the mice was assessed with the open field and the Morris water maze tests. In the first, the movements of PD mice focused in the central region, in contrast to the more peripheral presence of mice treated with mCu‐PDA/SOD. However, in *Cx3cr1* ^CKO^ PD mice, treatment of mCu‐PDA/SOD could not significantly alter the activity distribution of mice (Figure 7C,E,F). Similar results were obtained in the Morris water maze, where mCu‐PDA/SOD‐treated mice swam faster and showed more target occupancy than PD mice, and the inhibition of mCu‐PDA/SOD on PD behavioral disorder could be reversed by *Cx3cr1* knockout (Figure 7D,G,H).

To further investigate the effect of a *Cx3cr1* knockout in inhibiting pS129 formation and improving TH generation, we performed co‐immunofluorescence labeling for TH and pS129 in the SNpc of PD models. The images (Figure 7I) and corresponding quantitative results (Figure 7J) showed significant immunoreactivity for pS129 but less for TH in the SNpc of PD mice. In contrast, mCu‐PDA/SOD dramatically increased TH immunoreactivity, while decreasing that of pS129. However, the effects of mCu‐PDA/SOD on pathological symptom remission were partially inhibited by knocking out *Cx3cr1*. Furthermore, mCu‐PDA/SOD treatment significantly inhibited the expression of NLRP3, caspase‐1, and IL‐1β in the brain of PD mice, indicating that NLRP3 inflammasome‐mediated inflammatory response is associated with the therapeutic mechanism of mCu‐PDA/SOD. Moreover, the mCu‐PDA/SOD treatment could decrease the content of CX3CL1 and phosphorylated P65 (p‐P65; Figure 7K), suggesting an indispensable role of the microgel in regulating the chemokine axis and NF‐κB signaling, consistent with transcriptomic analysis (Figures 6B,C, and 7A). Importantly, the *Cx3cr1* knockout reversed the effect of mCu‐PDA/SOD, further indicating a key role of *Cx3cr1* in the effects of mCu‐PDA/SOD (Figure 7K). Collectively, mCu‐PDA/SOD could nurture dopaminergic neurons, while eliminating ROS, thus alleviating oxidative damage in nerve cells. Through the above synergies, mCu‐PDA/SOD inhibits the activation of the NF‐κB pathway through the CX3CL1/CX3CR1 axis, hindering inflammasome assembly and the release of inflammatory factors, thus decreasing neuroinflammatory damage (Figure 7L).

PD is a neurodegenerative disease with multiple pathogenesis, and its treatment requires a comprehensive strategy. The nanotherapy of PD has been paid much attention by neuroscientists and nanoscientists. For example, some research groups provide nanotherapeutic strategies such as removal of excess ROS,^[^ ^40^ ^]^ inhibition of α‐syn aggregation,^[^ ^41^ ^]^ and regeneration of neurons.^[^ ^42^ ^]^ However, most nanosystems have their own drawbacks. For example, liposome‐based nanosystems have limited BBB‐traversing ability and stability.^[^ ^43^ ^]^ Gold or mesoporous silica nanoparticles are non‐biodegradable and difficult to conjugate with biomolecules.^[^ ^44^, ^45^ ^]^ Hydrogel systems have some special properties that are more suitable for in vivo delivery and PD treatment. For example, encapsulating anti‐PD drugs, including activin B^[^ ^46^ ^]^ and L‐dopa,^[^ ^47^ ^]^ into hydrogels can simulate the extracellular matrix, benefiting cell adhesion and proliferation. In addition, hydrogels can elevate the efficiency of neuron regeneration after loading dopaminergic grafts in hydrogel systems.^[^ ^48^ ^]^ In particular, the study of microgel systems has become a new hot spot of nanodelivery in the central nervous system,^[^ ^49^ ^]^ and is expected to bring new solutions for PD treatment. However, the nano‐biological effects of microgel nanosystems on cell communications have rarely been explored, and this aspect is one of the vital but easily overlooked avenues in PD treatment. In this study, we tried to explore the field by exploring the signal pathways and key targets in microgel‐mediated cell communications. In the future, we will continue to explore the biological effects and mechanisms of nanomedicine in the communications between neurons, glial cells, and neural stem cells.

## 3 Conclusion

In summary, we developed a novel microgel system for PD management using functional monomers and free radical polymerization, achieving brain enrichment and regulation of the interaction between nerve cells concurrently. In the microgel system, the PDA and imidazole groups could coordinate with Cu ions, improving substrate transport and forming a catalytic module with its SOD‐ and CAT‐like activities. After traversing the BBB and accumulating at the brain lesion site, PDA can be degraded into DA which fosters neurogenesis, further weakening the production and secretion of CX3CL1 in dopaminergic neurons. The above processes, combined with the antioxidant effect of the microgel system, inhibited activation of the NF‐κB‐NLRP3 pathway and inflammasome production in microglia, relieving neuroinflammation and pathological symptoms of PD. Therefore, this study provided fundamental insights into the biological effects of microgel systems interfering with communications between neurons and microglia, highlighting the key role of the chemokine axis in the management of neuroinflammation, and informing the design and application of functional nanodrugs for neuroinflammatory regulation in PD.

## 4 Experimental Section

The detailed experimental processes are available in the Supplementary Information.

## Conflict of Interest

The authors declare no conflict of interest.

## Supporting information



Supporting Information

## Data Availability

The data that support the findings of this study are available from the corresponding author upon reasonable request.
