# Supplementary material for: Functional Monomers Equipped Microgel System for Managing Parkinson's Disease by Intervening Chemokine Axis‐mediated Nerve Cell Communications
Source: Adv Sci (Weinh). 2024 Dec 25;12(7):2410070. doi: 10.1002/advs.202410070 (PMC11831437; doi:10.1002/advs.202410070)
Supplement: Supplementary file 1 — Supporting Information [file ADVS-12-2410070-s001.docx]

**Supplementary Information**

**Functional monomers equipped microgel system for managing Parkinson’s disease by intervening chemokine axis-mediated nerve cell communications**

*Lin Jiang*^#^*, Xu Zhang*^#^*, Shun Wang*^#^*, Jiangkuan Zhang, Junyang Chen,* *Jiachuan Lu, Liting Yao, Weiwei Jin*, Nan Li*, Qing Li**

Dr. L. Jiang, Miss. L. Yao, and Prof. W. Jin

College of Life Sciences, China Jiliang University, Hangzhou 310018, China.

Email: wwjin0722@cjlu.edu.cn

Miss. X. Zhang, Dr. S. Wang, Mr. J. Zhang, Mr. J. Lu, Prof. N. Li, and Prof. Q. Li

Department of Neurology, The Second Affiliated Hospital of Zhengzhou University, Zhengzhou University, Zhengzhou 450052, China.

Email: linan@zzu.edu.cn; [sahlq@zzu.edu.cn](mailto:sahlq@zzu.edu.cn)

Dr. J. Chen

School of Life Sciences, Zhengzhou University, Zhengzhou 450001, China.

^#^ These authors contributed equally to this work.

*** Corresponding authors.

**Experimental Section**

**Materials.** SOD was purchased from Sigma-Aldrich. Vinyl imidazole (VIM), 2-Methacryloyloxy ethyl phosphorylcholine (MPC), Poly(ethylene glycol) methyl ether methacrylate (PEGMA), Ethylene dimethacrylate (EDGMA), N-hydroxy succinimide (NHS), 1-(3-Dimethylaminopropyl)-3-ethylcarbodiimide hydro (EDC), dopamine (DA) L-ascorbic acid sodium (L-ASC) ammonium persulphate (APS) acrylic acid (AA) were purchased from J&K Scientific. Dulbecco’s modified Eagle’s medium (DMEM) and fetal bovine serum (FBS) were obtained from Gibco (California, America). Thiazolyl blue tetrazolium bromide (MTT) was purchased from Sigma-Aldrich. SH-SY5Y cells and BV2 cells were purchased from Pricella Life Technology Co., Ltd (Wuhan, China). 1-methyl-4-phenyl-1,2,3,6-tetrahydropyridine (MPTP) hydrochloride was purchased from the Sigma-Aldrich. C57BL/6-*Cx3cr1^em1Cflox^*/Cya mice and Tmem119-2A-CreERT2 mice were purchased from Cyagen (Suzhou, China). Malondialdehyde (MDA) and reactive oxygen species (ROS) commercial assay kit were purchased from Nanjing Jiancheng Bioengineering Institute (Nanjing, China). All other reagents were of the highest grade commercially available and used as received.

**Synthesis of mSOD, mPDA/SOD, mCu-PDA/SOD.** 30.0 mg SOD, 153.0 mg NHS and 193.0 mg EDC were dissolved in 10 mL deionized water (DI-water), and 100.0 mg AA was added into the solution. After stirring overnight at room temperature (RT), the solution was dialyzed by dialysis membranes with a cut-off value of 3500 Da in for 72 h, and change DI-water every 24 h. Finally, freeze-dried the solution to get SOD with double bond.

30.0 mg SOD with double bond, 50.0 mg PEGMA, 29.5 mg MPC, 9.4 mg VIM, and 19.8 mg EDGMA were dissolved in 10 mL DI-water, then 150 μL 10% APS solution was added into the solution. After stirring at 40 °C for 4 h, the solution was dialyzed by dialysis membranes with a cut-off value of 3500 Da in DI-water for 72 h, and changed DI-water every 24 h. Finally, freeze-dried the solution to get mSOD.

30.0 mg SOD with double bond, 50.0 mg PEGMA, 29.5 mg MPC, 9.4 mg VIM, and 19.8 mg EDGMA were dissolved in 9 mL DI-water, then 150 μL 10% APS solution was added into the solution. After stirring at 40 °C for 1 h, 1 ml 0.1 mM DA solution was added into the mixture for 3 h. Finally, the product was dialyzed by dialysis membranes with a cut-off value of 3500 Da in DI-water for 72 h, and changed DI-water every 24 h. Finally, freeze-dried the solution to get mPDA/SOD.

150.0 mg mPDA/SOD was dispersed in 8 mL DI-water, then 1 mL 30 mg/mL CuCl_2_ and 1 mL 45 mg/mL L-ASC were added in the mPDA/SOD solution. After stirring at RT for 10 min, the product was dialyzed by dialysis membranes with a cut-off value of 3500 Da in DI-water for 72 h, and changed DI-water every 24 h. Finally, freeze-dried the solution to get mCu-PDA/SOD. Cu ions coordinates under neutral conditions.

**Characterizations.** Transmission infrared (FTIR) spectra of mCu-PDA/SOD were measured by using a Fourier transform infrared spectrophotometer 8400 (Nicolet is5 from Thermo Fisher). Powder X-ray diffraction (PXRD) patterns were collected on a PANalytical B.V. Empyrean powder diffractometer in which raw data were collected from 5 to 80° at a scan rate of 10° min^-1^. Scanning electron microscope (SEM), was performed on a Hitachi SU4800 instrument. Thermogravimetric analysis (TGA) was carried out using a Mettler TGA Thermogravimetric Analyzer and data were collected from 30 to 800 °C at a scan rate of 20 °C min^–1^. X-ray Photoelectron Spectroscopy (XPS) analysis of the samples was collected on a Thermo Fisher ESCALAB 250. The density functional theory (DFT) was performed using Materials Studio (MS). The elemental core and valence electrons were represented using the CASTEP method.The Perdew-Burke-Ernzerhof generalized gradient approximation (GGA-PBE) functional was employed to estimate the exchange-correlation potential energy. The SOD microgels and Cu-based SOD microgels were evaluated using transmission electron microscopy (TEM, Talos, F200x). The content of SOD in microgel was determined using the Nanodrop 2000c (ThermoFisher Scientific) through Nano-Drop 2000 program, and the absorbance was measured at 280 nm wavelength.

**Cu content detection.** Inductively coupled plasma-mass spectrometry (ICP-MS, Perkin Elmer Elan 6100) was used to determine the iron concentration of mCu-PDA/SOD. 1 mg of the sample (after treated in pH=5 or pH=7 PBS solution for 1 h and dialysis to purify) was added to a 1.5 mL centrifuge tube and stored with HNO_3_ (1 g, ultrapure). Before ICP-MS analysis, dilute the sample 10 times with deionized water. The measured values reported are the average of three technical replicates.

**SOD-like enzyme activity detection.** The O_2_^•−^ elimination rates of the microgels were used to calculate their SOD-like activity. Cytochrome C was used as an indicator for O_2_^•−^, which was produced by the xanthine-xanthine oxidase system. The control system consisted of 0.5 mL xanthine, 0.5 mL cytochrome C, 0.2 mL xanthine oxidase, and 1.8 mL PBS buffer. The O_2_^•−^ concentration of the control system was determined by measuring the increase in absorbance at 550 nm (ΔA1) of cytochrome C using an ultraviolet spectrophotometer for 1 minute. After adjusting ΔA1 to 0.025, microgels with different concentrations (0.000417 ⁓ 0.125 mg mL^-1^) were individually added to the control system, and the increase in absorbance at 550 nm (ΔA2) of cytochrome C was recorded for 1 minute. The O_2_^•−^ elimination rate of nanozymes was calculated using the formula: (ΔA1 - ΔA2) / ΔA1 * 100%.

**CAT-like enzyme activity detection.** UV-Vis absorption spectra were used to test the CAT-like properties by measuring the absorbance of the hydrogen peroxide (H_2_O_2_) in the absence and presence of nanoparticles. In a typical analytical method, hydrogen peroxide solution (180 μL, 50 mM) and nanoparticles (free SOD, mSOD, mPDA/SOD, mCu-PDA/SOD, and mCu/SOD) (20 μL) were mixed in the colorimetric dish and the absorbance at 734 nm was measured at different reaction time. The mixture of ABTS (7 mM) dissolved in ultrapure water and potassium persulfate (2.45 mM) was kept in the dark for 12 h before use in order to form the stable blue–green cationic radical ABTS^+^, namely the ABTS solution. The different nanoparticles (free SOD, mSOD, mPDA/SOD, mCu-PDA/SOD, and mCu/SOD) were mixed with a stable ABTS solution, and the absorbance value was recorded at 734 nm. The experiments described above were both performed in triplicate, and 70% ethanol was taken as control.

**Electrocatalytic performance detection.** Electrochemical tests were conducted on a three-electrode system of electrochemical workstation (Electrochemical Analyzer, CHI760E, Shanghai). First, nanoparticles (20 μL, 0.5 mg/mL) were deposited on the glassy carbon (GC) electrode. Then nafion solution (10 μL, 5 mg/mL) was added on modified GC electrode. Cyclic voltammetry (CV) measurements with scan rate of 100 mV/s were performed using the electrochemical workstation. The H2O2 reduction and Oxidation Reduction Reaction were tested with a Pt wire as the counter electrode and a saturated calomel electrode (SCE) as the reference electrode, and the electrolyte were N2-saturated PBS.

**Cell viability assay**. Cells were seeded into 96-well plates at a density of 8000 cells/well overnight. The cells were then incubated with different microgel systems for 6 h and/or with 2 mM MPP^+^ (or 100 ng/mL LPS) for 24 h. Afterwards, the cells were incubated with the addition of MTT (5 mg/mL, 20 μL) at 37 °C for another 4 h, following adding dimethyl sulfoxide (DMSO) to dissolve the formed formazan crystal. Finally, the absorbance at 492 nm was measured using a microplate reader (GF-M3000, Shandong, China).

**ROS content analyses.** SH-SY5Y cells were treated with microgel systems at the optimized concentrations (60 μg/mL) for 6 h and then incubated with MPP^+^ for 2 h in an incubator. The cells were stained with 5 μM DCFH-DA for 30 min at 37 °C. Afterwards, images were acquired using a laser confocal microscope (FV1200, Olympus, Japan).

**Inflammatory factors detection** The BV2 cells were seeded into 6-well plates at a density of 2.0 × 10^5^ cells per well and incubated at 37 °C overnight. The cells were then incubated with microgel systems (60 μg/mL) for 6 h, and exposed to 100 ng/mL LPS for 6 h. The levels of IL-6 and IL-10 released in the media were determined through ELISA assays according to the manufacturer’s protocols.

**Isolation of microglia.** The mice midbrain tissue was separated and homogenized by adding 5 mL PBS in a homogenizer, and then the homogenized tissues were filtered by a 70 μm cell filter. The obtained cells masses were centrifuged (1000 g, 10 min) at 4 ℃ and then re-suspended with 5 mL 70% Percoll. After centrifugation at 800 g for 30 min, the myelin-rich upper layer was removed, the middle layer was obtained and mixed with 40 mL pre-cooled PBS, and then centrifugation (1400 g, 5 min) at 4 ℃ to get microglia-rich cell masses.

**BBB permeability analysis *via* a BBB model.** BEnd.3 cells in combination with transwell chambers were used to prepare a BBB model *in vitro*. Subsequently, the SH-SY5Y cells (lower chamber) and bEnd.3 cells (upper chamber) were co-cultured for 5 days until a tight junction (transepithelial electrical resistance (TEER) of 200 Ω cm^2^) was formed by bEnd.3 cells. The culture media was replaced with FITC-labeled microgel systems to examine the BBB permeability. 24 h after incubation in dark, the fluorescence intensity was quantified *via* Image J., and the cells in the lower chamber (SH-SY5Y cells) were collected and the images were obtained using a laser confocal microscope. The TEER was measured at 0, 4, 8, 12, 24 hours respectively after microgel systems incubation.

**Experimental animals.** All animal experiments were approved by the Institution Animal Ethics Committee of Zhengzhou University (license No. ZZU-LAC20230705[12]). Five-week-old C57BL/6 mice (SiPeifu Biotechnology Co., Ltd., Beijing, China) were housed in cages in a room with controlled temperature and humidity. All mice were allowed free access to water and food. Before experimentation, all mice were allowed to adapt to the animal house for 7 days. The house lighting was adjusted to 12 h light/dark cycles to simulate the natural environment of day and night.

**Parkinson's Disease (PD) Model.** For MPTP-induced PD models, the mice were subjected to rotarod performance testing on the rotation rod and those exhibited behavioral consistence were selected for further experiments. Mice were then intraperitoneally injected with MPTP (35 mg/kg/day for 7 consecutive days) to induce PD-like phenotype. For α-syn PFF-induced PD models, the α-syn PFF were sonicated for 10 min before usage. Next, the α-syn PFF (5×10^-3^ μg mL^-1^) were stereotactically delivered into the ST (+ 2.0 mm medial-lateral; + 0.2 mm antero-posterior; + 2.6 mm dorsoventral from bregma) of each mouse at a rate of 0.3 μL/min. Then, the needle was maintained in place for an additional 5 min and then slowly removed from the brain accompanying with monitor of wound healing and recovery after surgery. They were then randomly divided into 5 groups: MPTP (or α-syn PFF), mSOD, mPDA/SOD, mCu-PDA/SOD^-^, and mCu-PDA/SOD for subsequent treatment efficacy and mechanistical examinations. Healthy mice were assigned to the control group. mSOD, mPDA/SOD, mCu-PDA/SOD^-^, and mCu-PDA/SOD (10 mg/kg/day) were intravenously injected into mice every other day for 5 times after MPTP (or α-syn PFF) administration.

**Distribution of microgel systems in the brain and major organs.** Distribution of the microgel systems in the brain was determined in mice with PD-like phenotype. Briefly, mice with PD-like phenotype were injected with microgel systems (10 mg/kg) through the tail vein. 24 h after the injection, the mice in each group (n = 6) were sacrificed and major organs (heart, liver, spleen, lung, kidney, and brain) were collected. The harvested tissues were weighed and homogenized to determine the percentage of injected dose per gram of tissue (%ID g^-1^) using ICP-MS (Aglient 7800, China). To examine the distribution of the microgel systems in the SNpc and ST of the brain, microgel systems with or without fluorescence labeling (10 mg/kg) were injected into mice through the tail vein. Then, the mice in each group (n = 6) were sacrificed to collect brain tissues. The tissues for ICP-MS were weighed and homogenized to determine the percentage of injected dose per gram of tissue (%ID g^-1^) using ICP-MS. Meanwhile, the tissues for TEM analysis were fixed with 2.5% glutaraldehyde for 24 h and examined under a TEM (Hitachi H-7650, Japan). Additionally, the tissues for slice analysis were immersed in 4% paraformaldehyde, embedded in paraffin, and photographed *via* panoramic section scanner (Pannoramic, DHISTECH, Hungary).

**Open field test.** The open field test was performed 5 days after administration of microgel systems for evaluating autonomous behavior, exploratory behavior, and tension of experimental mice in novel environments. The apparatus (RWD 63041) was 50 × 50 × 40 cm. Each mouse (n = 6) was placed in the designated place and its motion path was recorded during the subsequent 5 min *via* SMART V3.0 software, and the time proportion in central zone, time proportion in peripheral zone and average speed were also recorded.

**Rotarod test.** The rotarod test was performed 5 days after administration of microgel systems. For the test, the untreated mice were pretrained on a fatigue rotating rod (ZB-200, Cheng Du, China) at a rotation speed of 8, 16, 24 rpm and constant acceleration from 4 to 44 rpm for 5 min, respectively. Afterwards, the treated mice (n = 6) were tested on a rotating rod at a speed of 20 rpm for 2 min for three times. The latency of each mouse on the rod and the total drops were recorded, and the final score was recorded as the average time of the three tests.

**Histochemical analyses.** Excised brain specimens of the mice with PD-like phenotype were fixed in 4% formaldehyde for 72 h. Thereafter, the SNpc and ST tissues were collected and embedded in paraffin. The tissues were sectioned into 8 μm ultra-thin slices (Leica UC7, Germany) which were stained for subsequent histological analysis. The sections were placed in the EDTA (pH = 9.0) solution for antigen retrieval in a microwave oven set at a medium heat for 8 min to boil, followed by 8 min in a warm temperature, and in a medium heat for 7 min. The endogenous peroxidase activity was blocked by incubation with 3% H_2_O_2_ for 25 min followed by blocking with 3% BSA for 30 min at room temperature. The slices were incubated with antibodies against TH (GB12181, Servicebio, China), pS129 (ab51253, Abcam, dilution: 1:500), GFAP (GB12096, Servicebio, China), IBA-1 (GB13105-1, Servicebio, China), and CD206 (ab64693, Abcam, China) at 4 ℃ for 24 h. They were then treated with fluorescently- or HRP-labeled secondary antibodies for 30 min at room temperature. Finally, the sections were incubated with DAB reagent, subjected to hematoxylin counterstaining, and photographed under a microscope.

**Biosafety evaluation.** The C57BL/6 male mice were subjected to intravenously injection of mCu-PDA/SOD (10 mg/kg/day) or saline (control group) every other day for 5 times, and the mice in MPTP group were subjected to intraperitoneal injection with MPTP (35 mg/kg/day) for consecutive 7 days. Then, the blood samples of mice were collected and allowed to stand at room temperature for 30 min, after which the samples were centrifuged at 800 *g* for 20 min. Then, the supernatant serum samples were obtained, and hematological biochemical analyses including alanine transaminase (ALT), aspartate aminotransferase (AST), blood urea nitrogen (BUN) and creatinine (CRE) were detected. Some mice were sacrificed and organs (heart, liver, spleen, lung, and kidney) were gained. Afterwards, the tissues were fixed in 4 % paraformaldehyde for 48 h, followed by gradient dehydration with ethanol solutions. Then, the tissue blocks were embedded with melted paraffin wax after treating twice in xylene, and sliced *via* a microtome (RM2016, Leica, China), and the slices were stained with hematoxylin and eosin (H&E) and photographed by fluorescence microscope (XSP-C204, CIC, China). Additionally, the blood samples of other mice were collected at the appointed time and subjected to IgG, IgA, complement (C)3 and C4 detection.

**RNA sequencing analyses.**Trizol Reagent (Invitrogen, USA) was used to obtain the total RNA in brain tissues, and Oligo (dT) magnetic beads were used to enrich mRNA with polyA structure among total RNA. All RNA was interrupted to fragments in 200 ~ 300 bp by ion-interruption. After the library was constructed, PCR amplification was applied to enrich the library fragments, and then the library was selected according to the fragment size, which was 450 bp. Afterwards, the total concentration and effective concentration of the library were detected through 2100 Bioanalyzer (Agilent, USA). After RNA extraction, purification, and library construction, the library was subjected to paired-end sequencing using the Next-Generation Sequencing (NGS) based on Illumina HiSeq X10 (Illumina, San Diego, USA). For bioinformatics analysis, TopGO (http://www.bioconductor.org/packages/release/bioc/html/RamiGO.html) was used for GO enrichment analysis. Besides, KEGG enrichment analysis was carried out and then annotated by KAAS (<https://www.genome.jp/tools/kaas/>).

**Antioxidant factors analyses.** The brain tissues of mice (n = 3) were cut into pieces and homogenized in physiological saline at room temperature. The concentration of the proteins in the homogenized samples was determined through the BCA protein assay. Finally, the ROS level, SOD, MDA, and GSH/GSSG activities were detected by the corresponding assay kits for ROS (E004, Nanjing Jiancheng Bioengineering Institute), MDA (A003, Nanjing Jiancheng Bioengineering Institute), GSH/GSSG (KA3779, Abnova) and SOD (50105ES60, Yeasen) with the manufacturer’s protocol of the assay kit.

***Cx3cr1* knockout mouse (*Cx3cr1*^cKO^).** Specific knockout of *Cx3cr1* gene in the brain of C57BL/6 mouse was achieved using the *Cre-Loxp* recombinant enzyme system. In C57BL/6-*Cx3cr1^em1Cflox^*/Cya mice (S-CKO-01949, Cyagen, China), the upper and lower reaches of the second exon of *Cx3cr1* were inserted into two Loxp locus in the same direction, and deletion of this region should result in the loss of function of the mouse *Cx3cr1* gene. We breeded the purchased C57BL/6-*Cx3cr1^em1Cflox^*/Cya mice mice with microglia-specific Cre mice (Tmem119-2A-CreERT2, Cyagen, China) with tamoxifen induction. The mice whose progeny were flox homozygous and carried Cre were identified as the controlled knockout mice that specifically knocked out microglia *Cx3cr1*, namely *Cx3cr1*^cKO^ mice.

**Morris water maze test.** A maze composed of four quadrants, a platform and a circular pool was used for the morris water maze test. The trajectories of mice (n = 6) were recorded by SMART V3.0 software. On the first 5 days, all mice were trained to reach the platform independently. After this process, formal experiments were performed. Briefly, each mouse was placed into the water opposite the platform to swim freely and the platform used in the training experiment was removed from the maze. The mean speed and time in the target zone (target of occupancy %) of each mouse were used to assess the spatial acuity of mice.

**Statistical analysis.** All statistical analyses were conducted using the GraphPad Prism 8.0.2. The criteria for significance were: **P* < 0.01, ***P* < 0.005, ****P* < 0.001, *****P* < 0.0001, ns (not significant). The differences in means between 2 groups were compared *via* Student’s *t*-test. For more than 2 groups, the outcomes were compared *via* one-way ANOVA (with Tukey’s post hoc correction for multiple comparisons). The specific statistical method and statistical analysis results for each experiment were listed in the corresponding figure legends.

**Supplementary Figures**


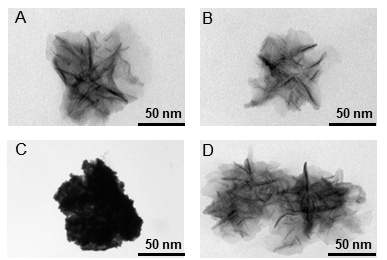


**Figure S1.** TEM images of (A) mSOD, (B) mPDA/SOD, (C) mCu-PDA/SOD and (D) mCu/SOD.

**Figure S2.** FTIR trace of mCu/SOD.

**Figure S3.** TGA curve of mCu/SOD.

**Figure S4.** Cu content (mass ratio) in mCu-PDA/SOD after treated in PBS solutions of pH = 5 or pH = 7, n = 3 independent experiments. Data represent the mean ± SD.

**Figure S5.** XRD curve of mCu/SOD.


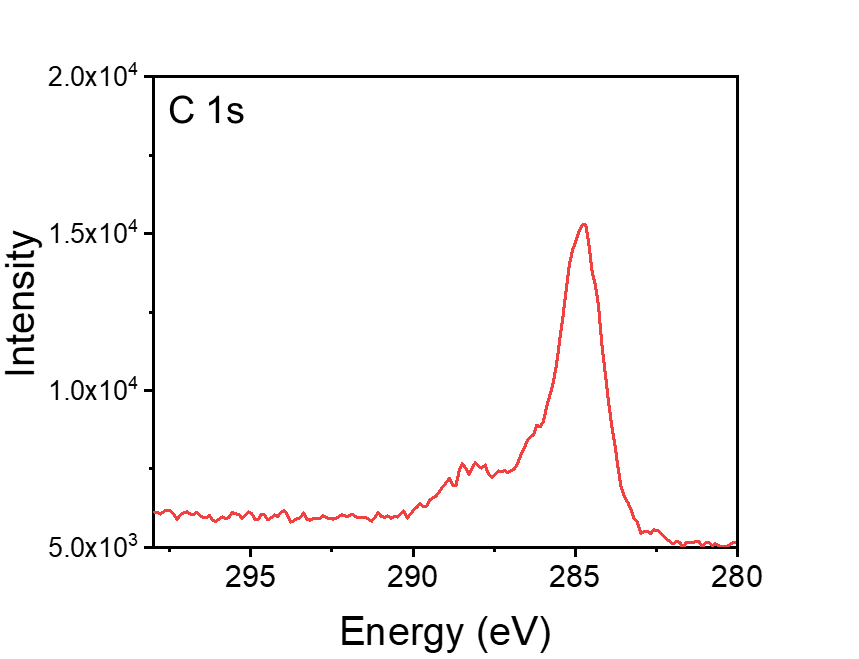


**Figure S6.** XPS C 1s spectrum of mCu-PDA/SOD.

**Figure S7.** SOD content (mass ration) in mCu-PDA/SOD and solution after reaction, n = 3 independent experiments. Data represent the mean ± SD.


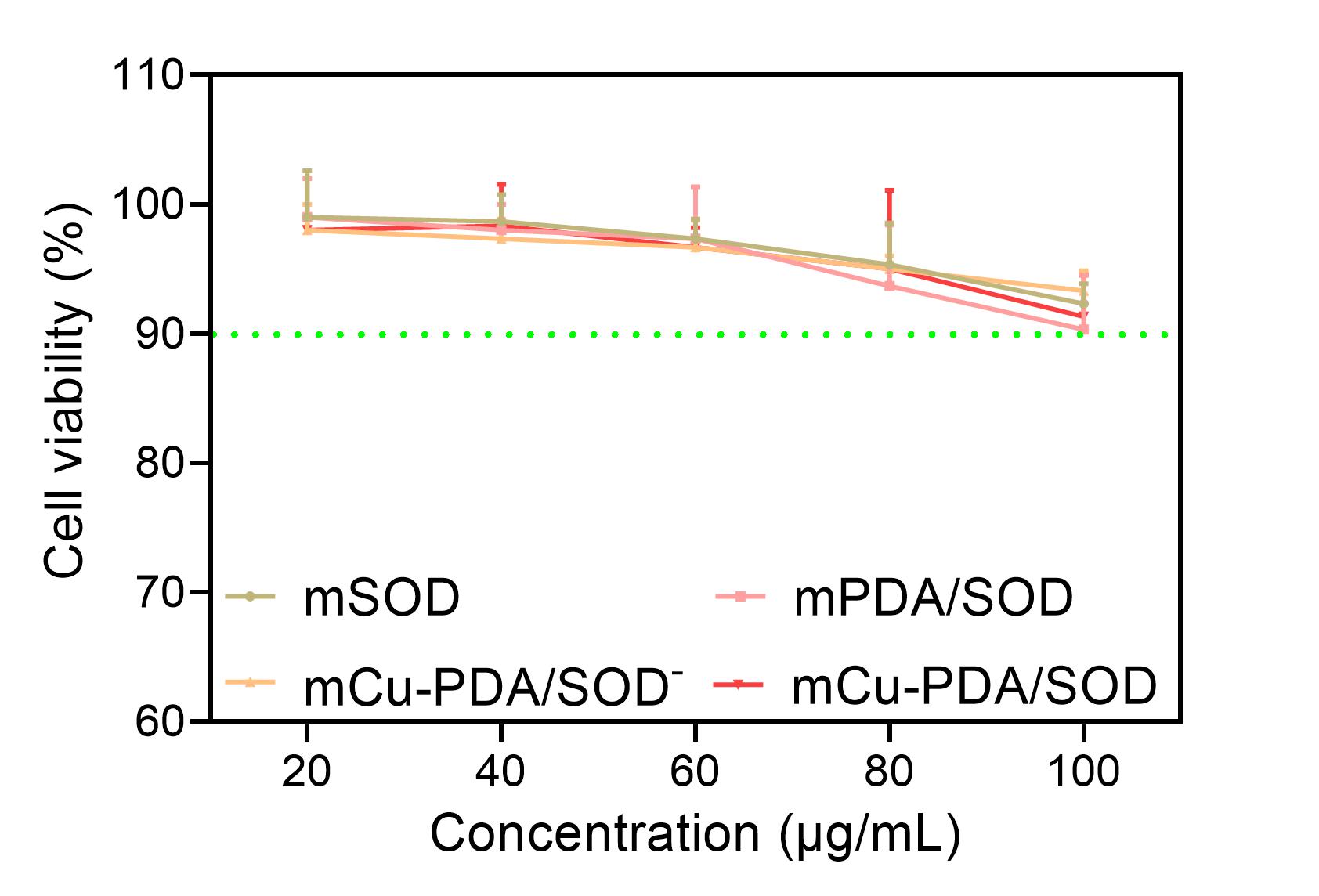
**Figure S8.** Cell viability of BV2 cells exposed to microgel systems of various concentrations, n = 3 independent experiments. Data represent the mean ± SD.


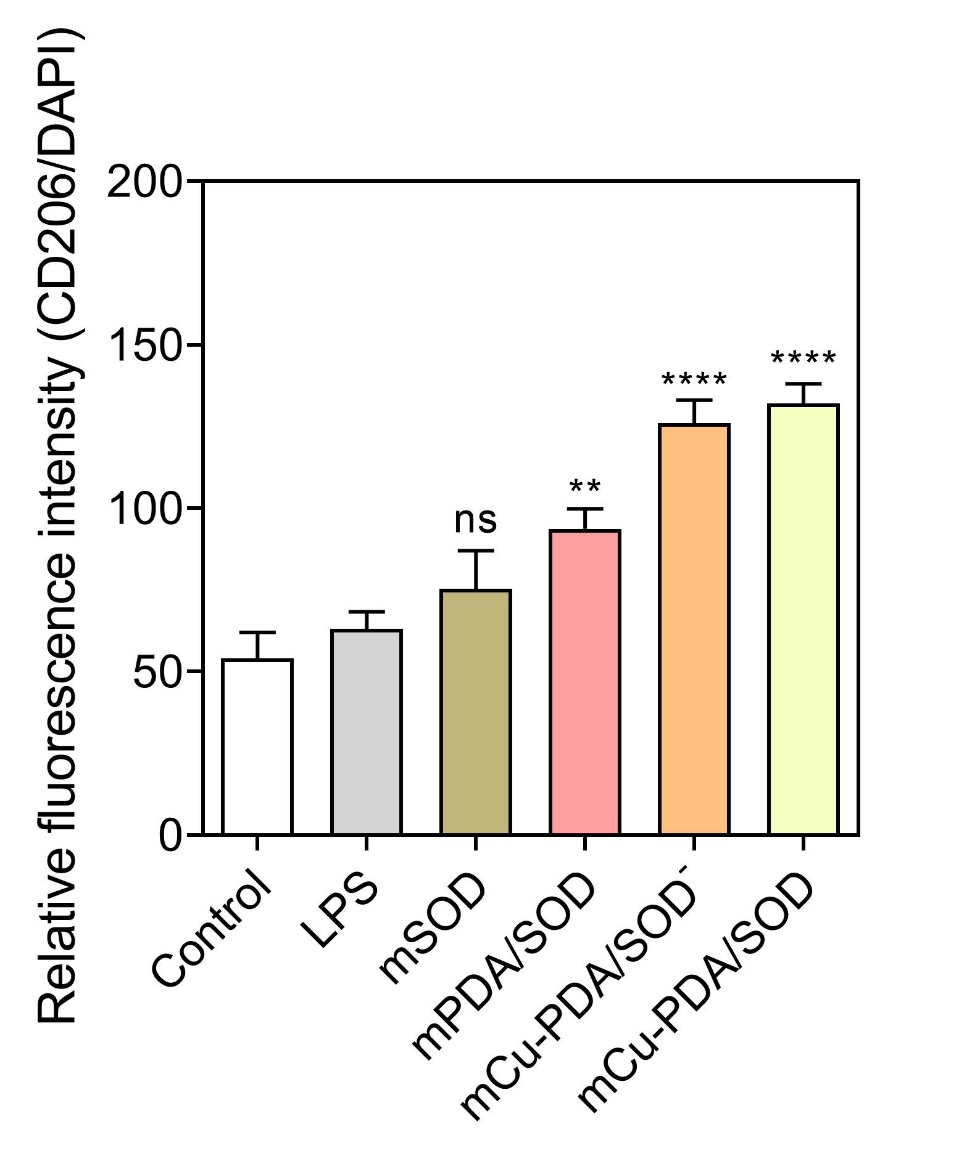
**Figure S9.** Quantitative analysis of CD206 immunofluorescence intensity in BV2 cells, n = 3 independent experiments. Data represent the mean ± SD. The statistical analyses were conducted using GraphPad Prism 8.0.2. The outcomes were compared *via* one-way ANOVA (with Tukey’s post hoc correction for multiple comparisons). ***P* < 0.005, *****P* < 0.0001, ns, not significant.


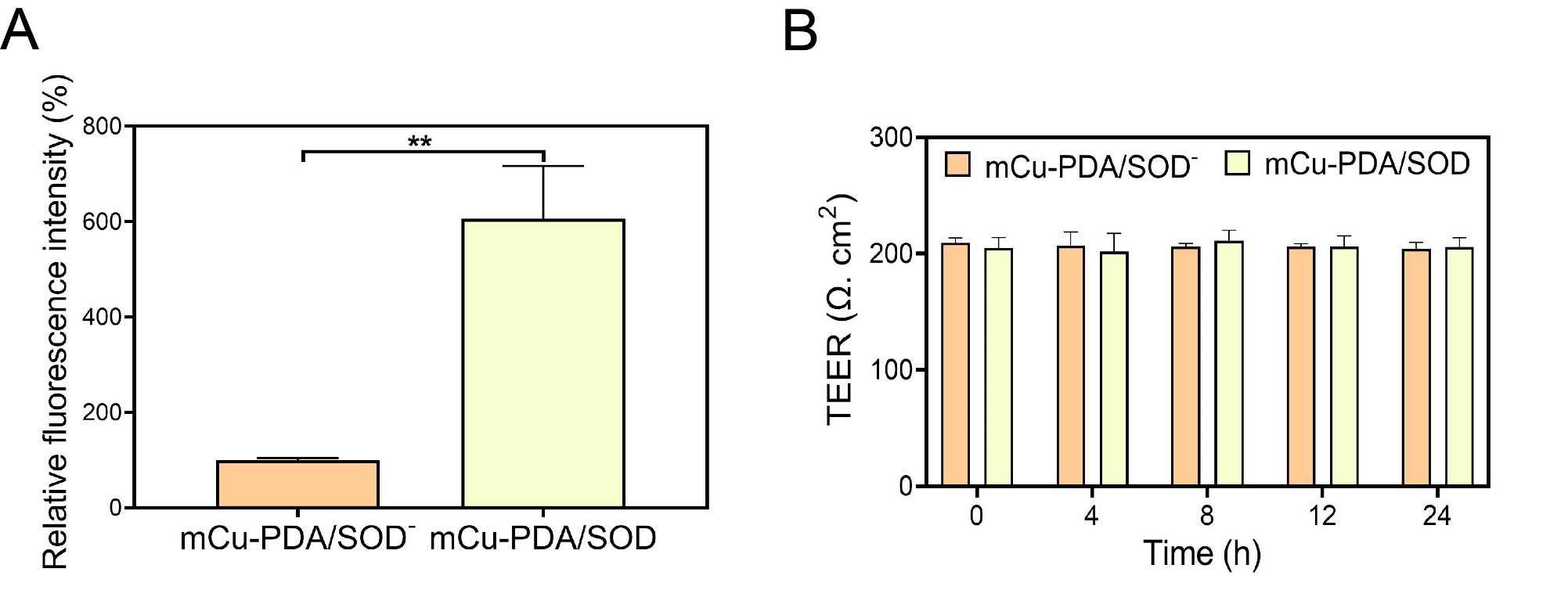
**Figure S10.** Quantitative fluorescence analysis showing the amount of microgel systems entering the cells, and the changes in the value of trans-endothelial electrical resistance (TEER) after treatment with the microgel systems for different time, n = 3 independent experiments. Data represent the mean ± SD. The statistical analyses were conducted using GraphPad Prism 8.0.2. The outcomes were compared *via* Student’s *t*-test. ***P* < 0.005.

**Figure S11**.
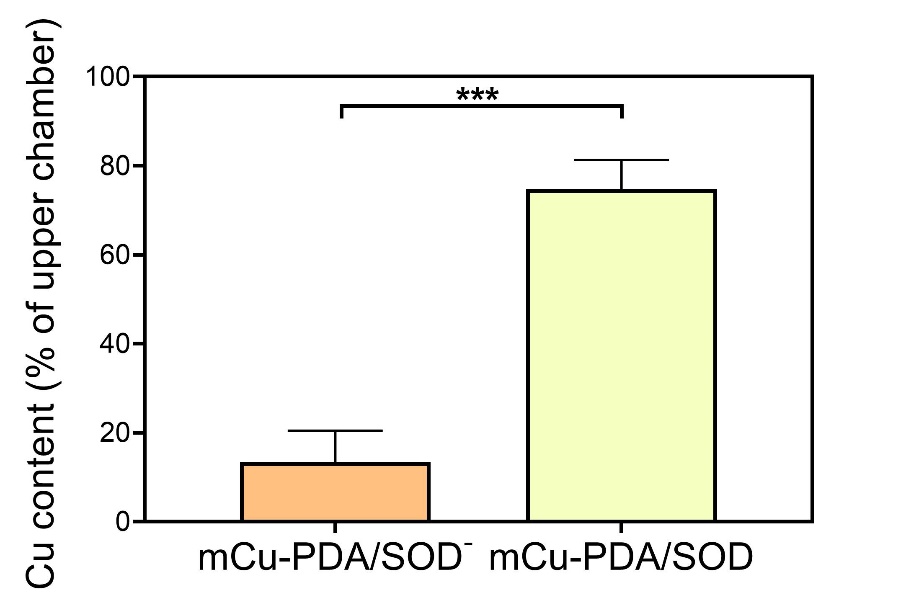
 The contents of mCu-PDA/SOD and mCu-PDA/SOD^-^ in the lower chambers of the traswell assay detected by ICP-MS, n = 3 independent experiments. Data represent the mean ± SD. The statistical analyses were conducted using GraphPad Prism 8.0.2. The outcomes were compared *via* Student’s *t*-test. ****P* < 0.001.

**
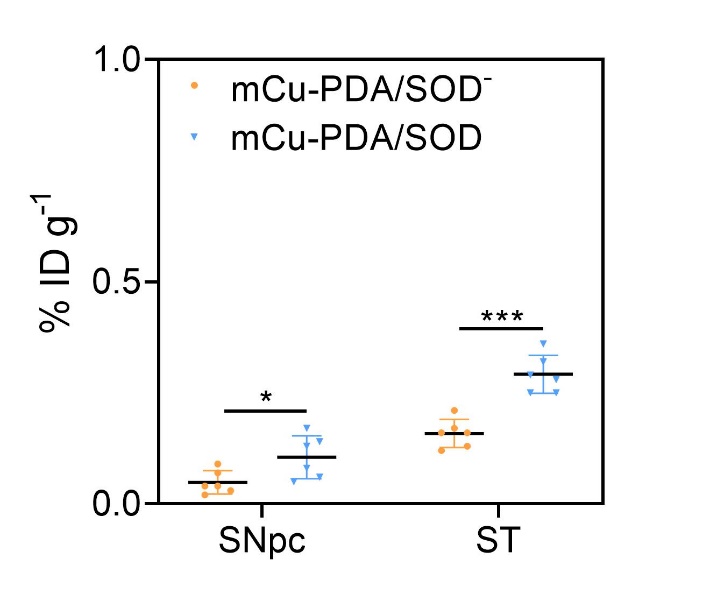
Figure S12.** The accumulations of mCu-PDA/SOD and mCu-PDA/SOD^-^ in the SNpc and ST of the brain detected by ICP-MS, n = 6 independent experiments. Data represent the mean ± SD. The statistical analyses were conducted using GraphPad Prism 8.0.2. The outcomes were compared *via* Student’s *t*-test. **P* < 0.01, ****P* < 0.001.

**Figure S13.**
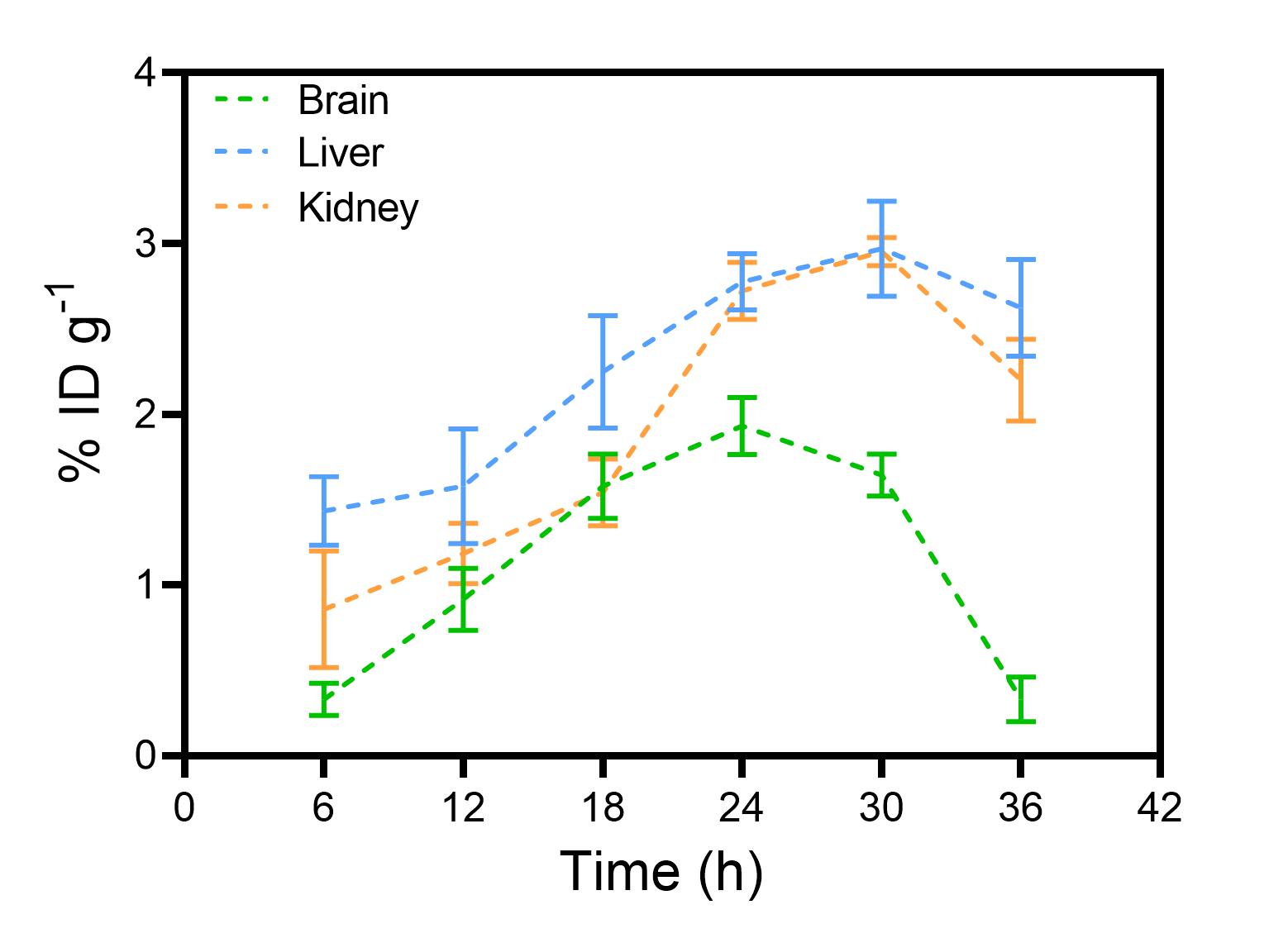
 ICP-MS analysis of the concentrations of mCu-PDA/SOD in the brain and the metabolism processes *via* the liver and kidney, n = 3 independent experiments. Data represent the mean ± SD.


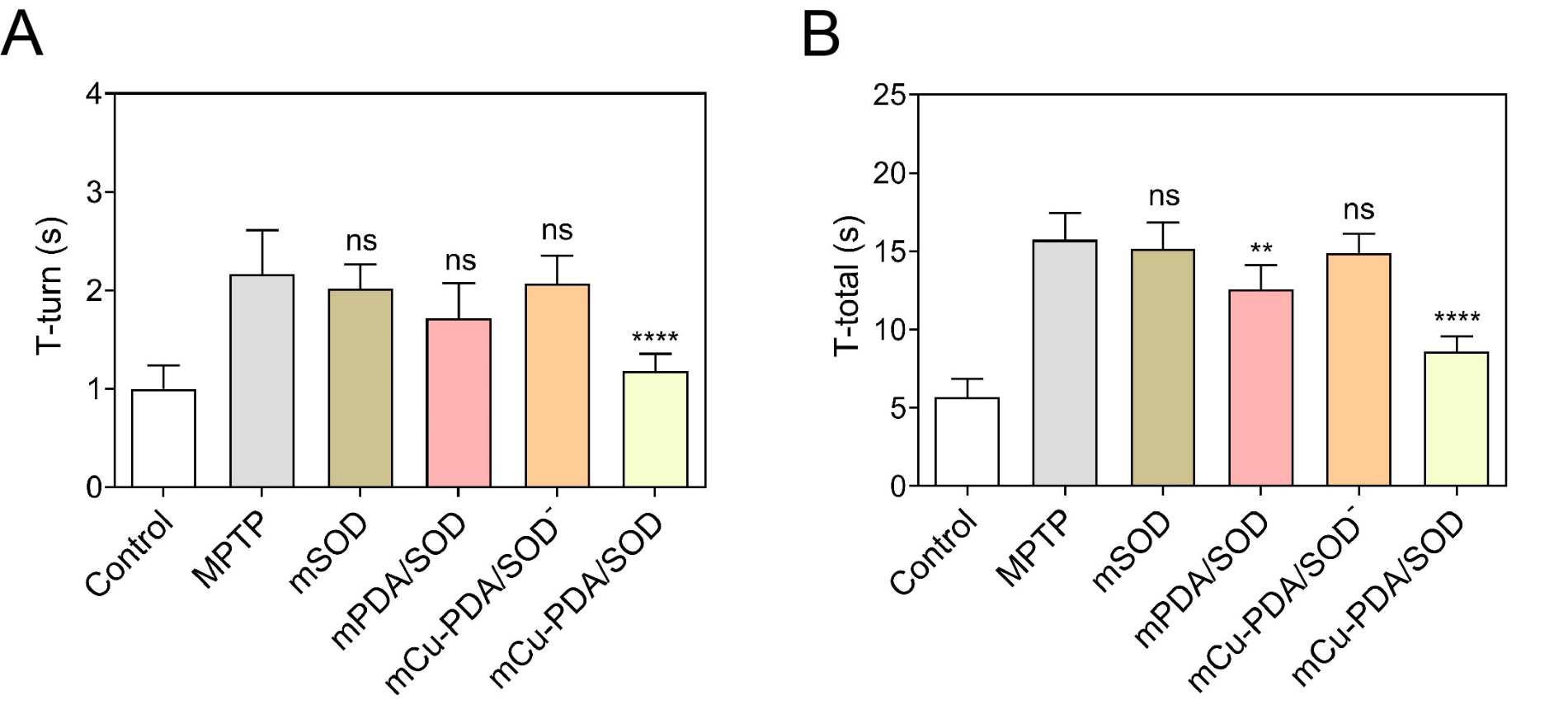
**Figure S14**. The main indicators in the pole test including (A) T-turn and (B) T-total, n = 6 independent experiments. Data represent the mean ± SD. The statistical analyses were conducted using GraphPad Prism 8.0.2. The outcomes were compared *via* one-way ANOVA (with Tukey’s post hoc correction for multiple comparisons). . ***P* < 0.005, *****P* < 0.0001, ns, not significant.


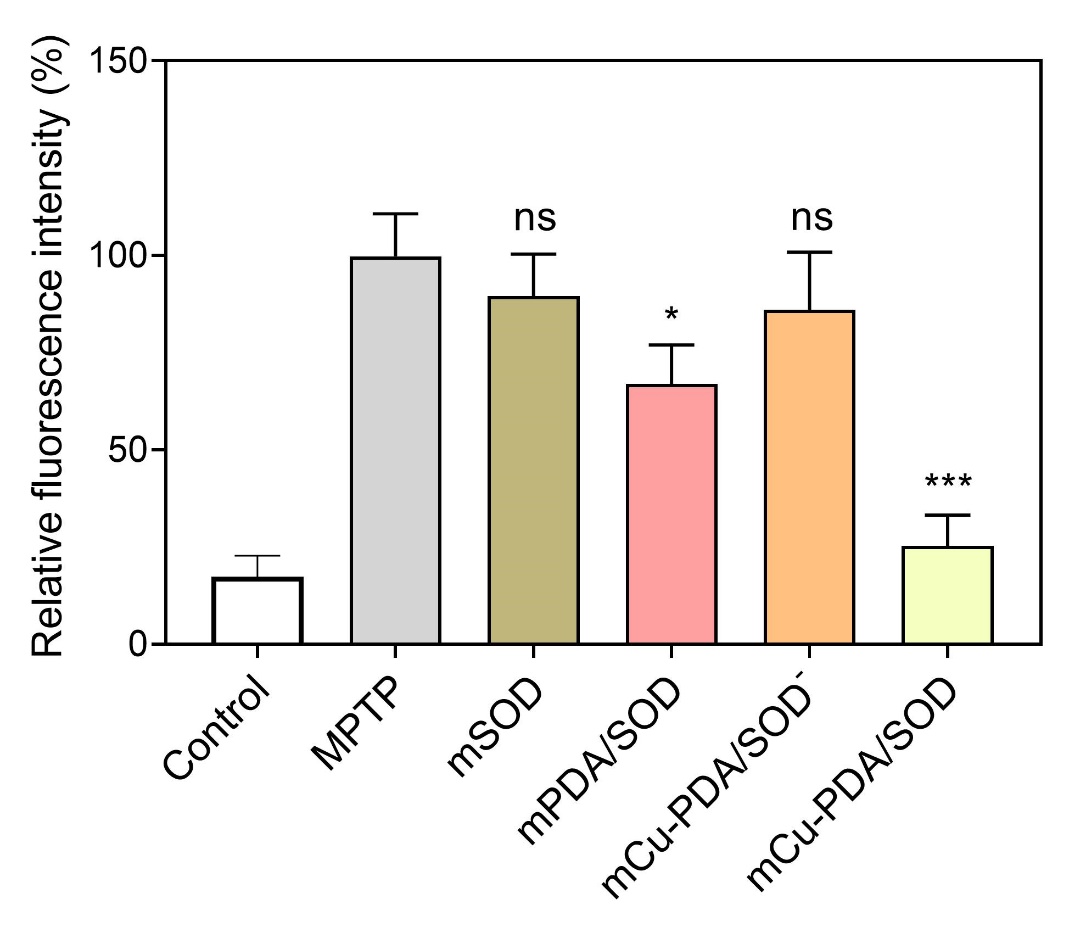
**Figure S15.** Quantitative analysis of the relative fluorescence intensity in the co-immunoreactivity staining of pS129 and TH, n=3 independent animals. Data represent the mean ± SD. The statistical analyses were conducted using GraphPad Prism 8.0.2. The outcomes were compared *via* one-way ANOVA (with Tukey’s post hoc correction for multiple comparisons). **P* < 0.01, ****P* < 0.001, ns, not significant.


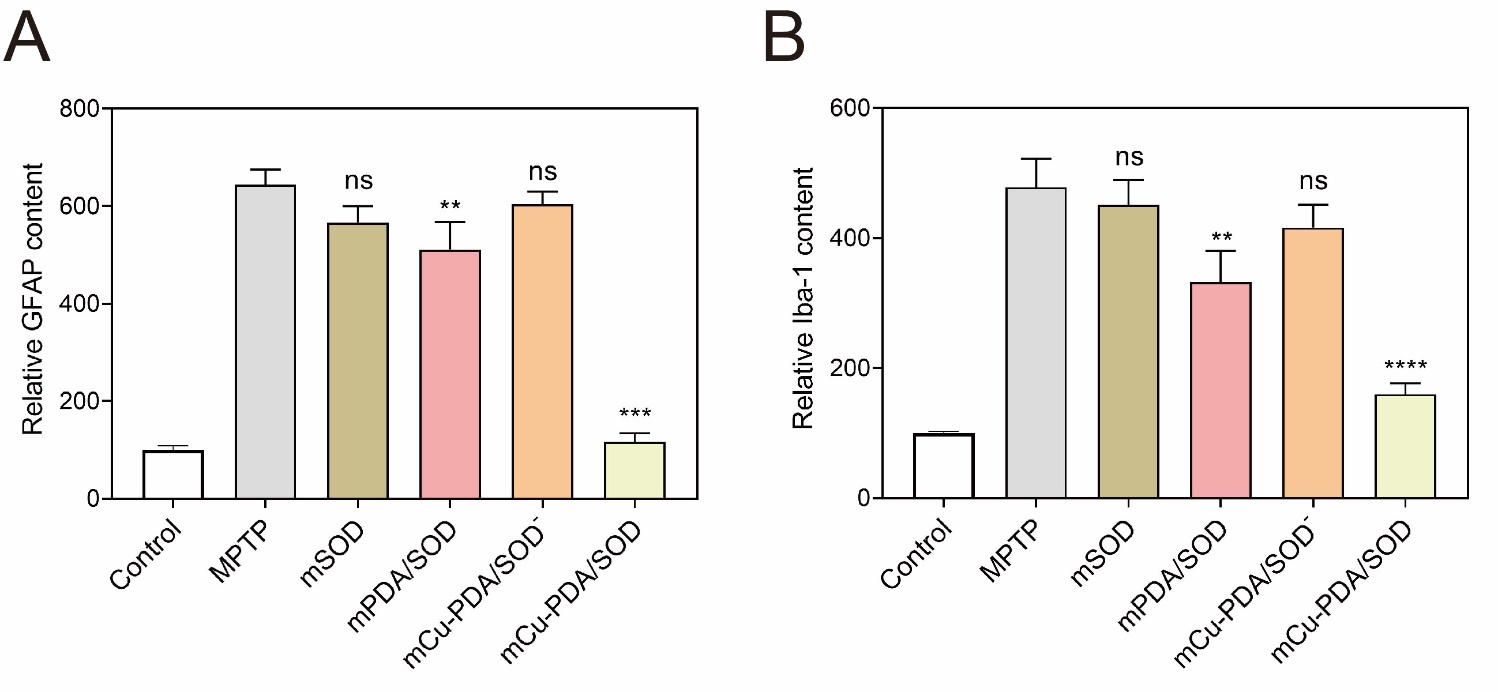
**Figure S16.** The relative (A) GFAP and (B) Iba-1 contents compared with the control group detected by the quantitative analysis of fluorescence intensity, n=3 independent animals. Data represent the mean ± SD. The statistical analyses were conducted using GraphPad Prism 8.0.2. The outcomes were compared *via* one-way ANOVA (with Tukey’s post hoc correction for multiple comparisons). ***P* < 0.005, ****P* < 0.001, *****P* < 0.0001, ns, not significant.

**
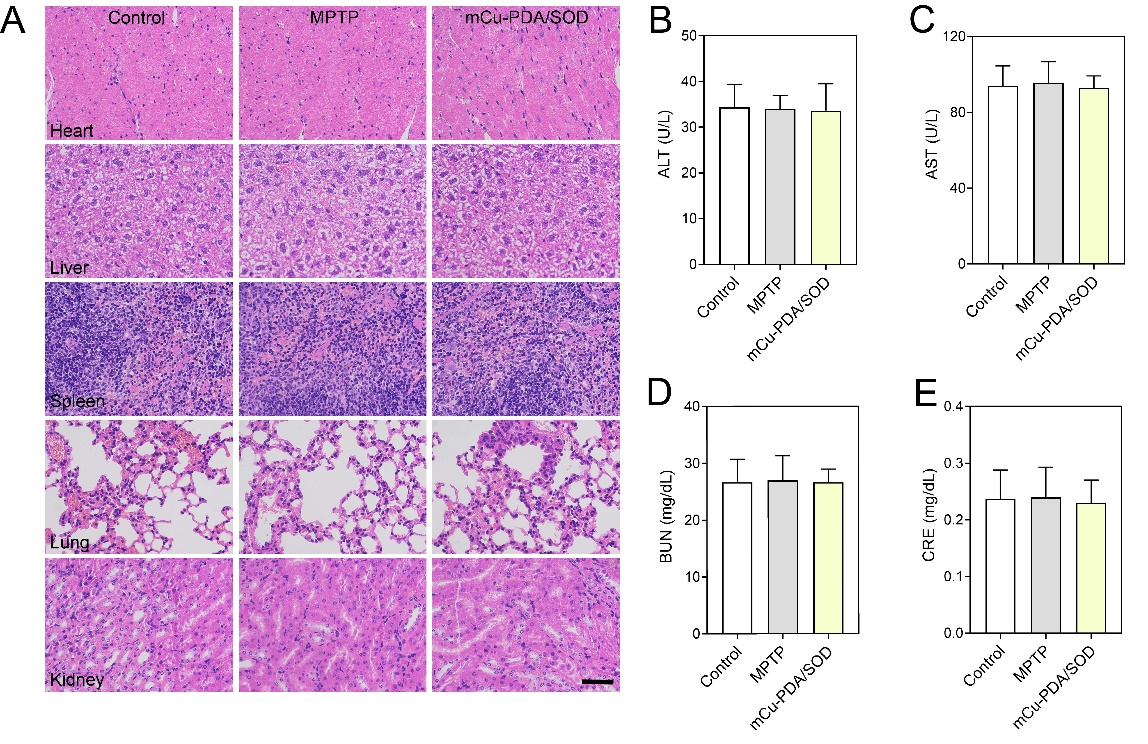
Figure S17.** The toxicity analyses of the major organs and serum parameters from the mice post-treatment with mCu-PDA/SOD. (A) Hematoxylin and eosin staining of the liver, lung, kidney, heart, and spleen in the mice. The scale bar is 50 μm. (B) Serum biochemical analyses of the PD mice treated with mCu-PDA/SOD. ALT, alanine transaminase; AST, aspartate transaminase (C); BUN, blood urea nitrogen (D); CRE, creatinine (E), n = 3 independent animals. Data represent the mean ± SD.


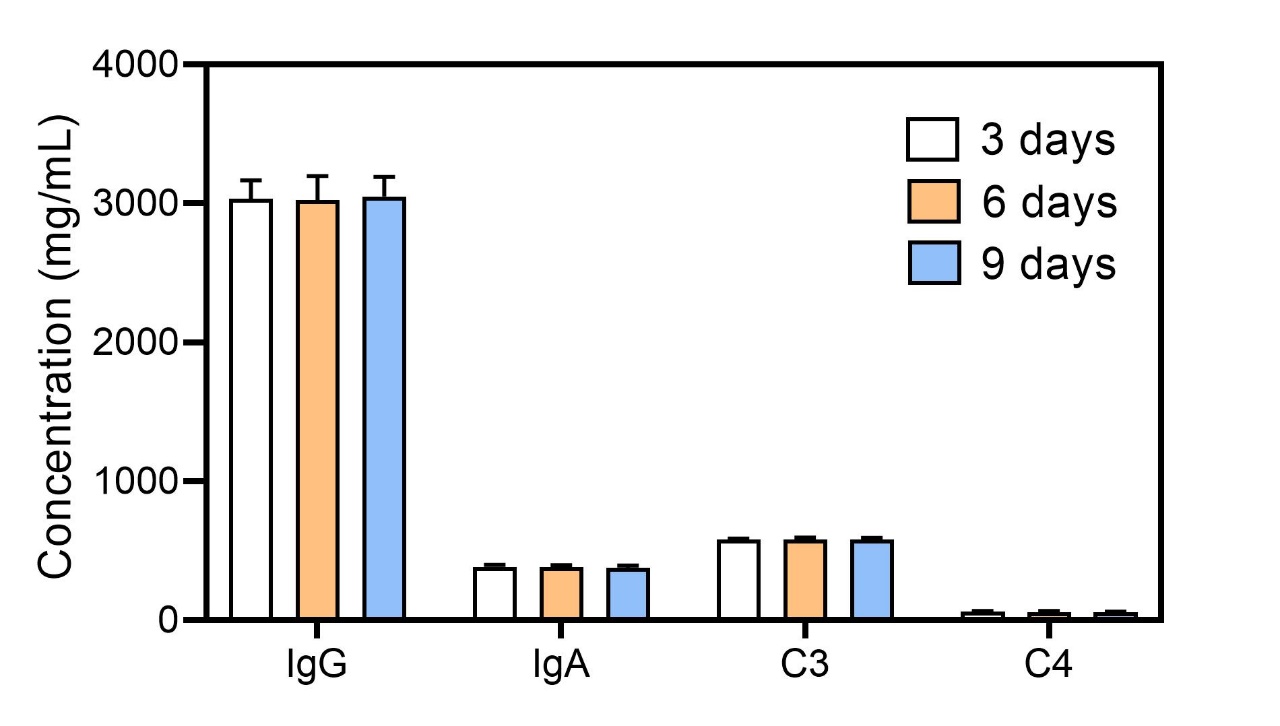
**Figure S18.** The concentrations of immune antibodies and complements (IgG, IgA, C3 and C4) at different times after mCu-PDA/SOD administration, n = 3 independent animals. Data represent the mean ± SD.


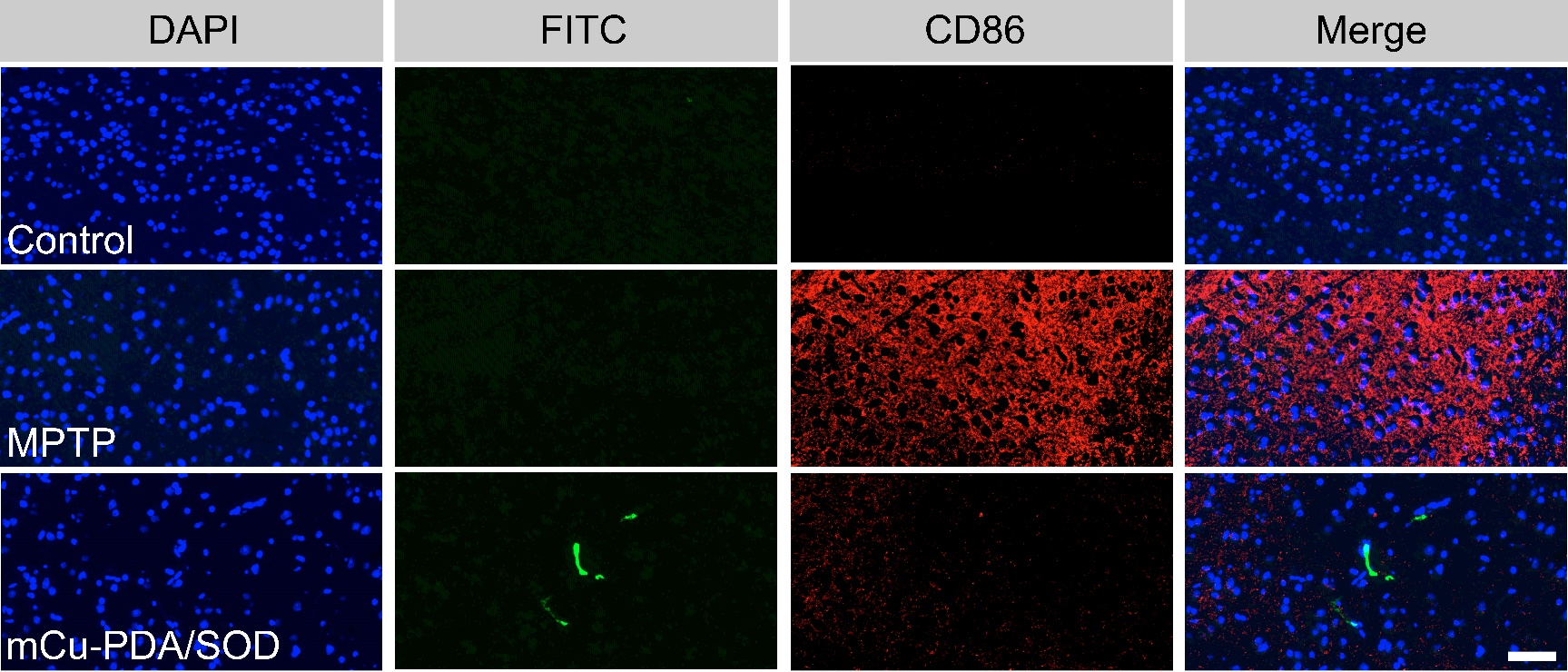
**Figure S19.** Immunofluorescence staining of CD86 in the SNpc of PD mice after treatment with microgel systems. The scale bar is 50 μm.

**Figure S20.**
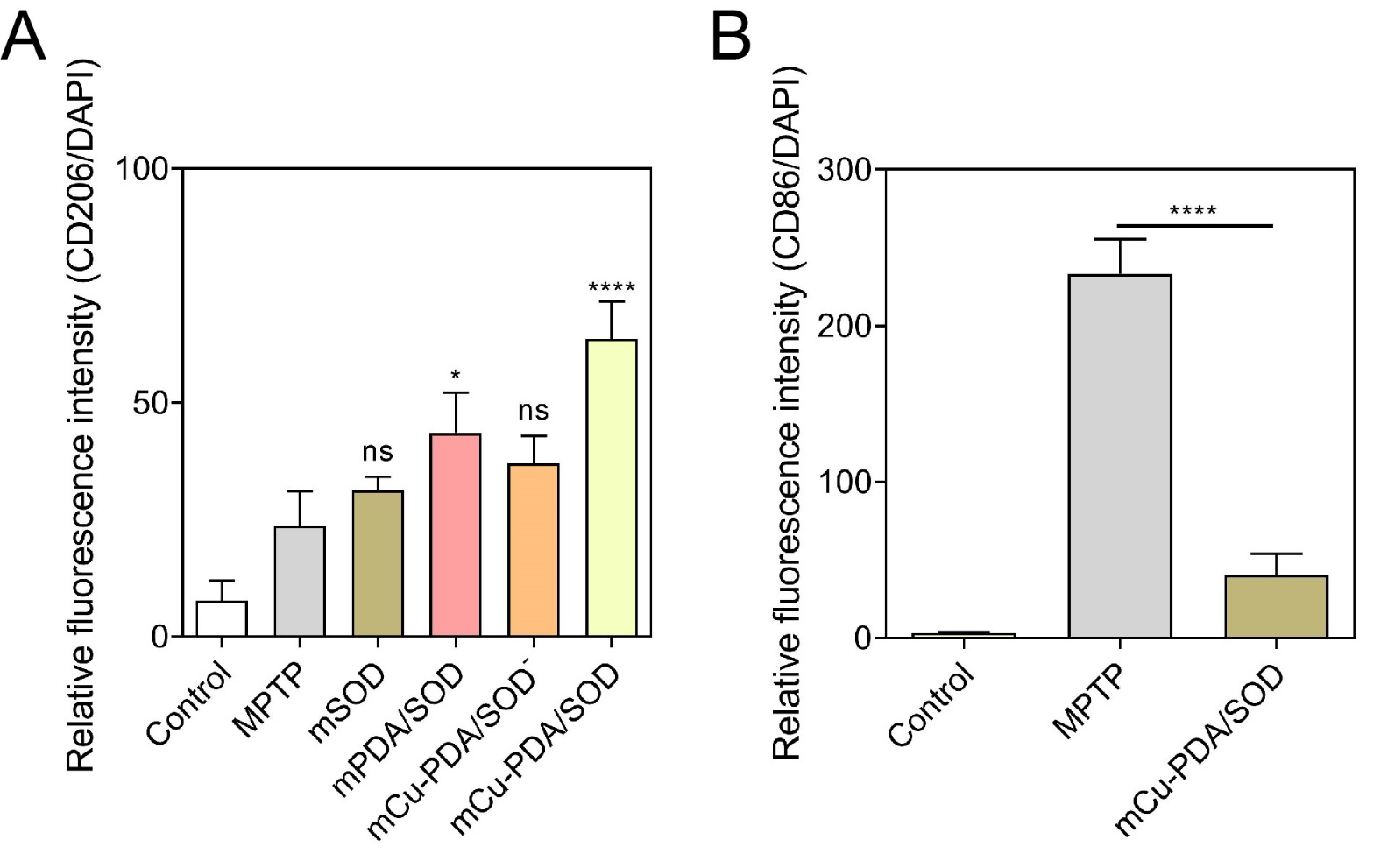
 Quantitative analysis of (A) CD206 and (B) CD86 immunofluorescence intensity in the SNpc of PD mice after treatment with microgel systems, n = 3 independent experiments. Data represent the mean ± SD. The statistical analyses were conducted using GraphPad Prism 8.0.2. The outcomes were compared *via* one-way ANOVA (with Tukey’s post hoc correction for multiple comparisons). **P* < 0.01, *****P* < 0.0001, ns, not significant.
